# ISR Modulators in Neurological Diseases

**DOI:** 10.2174/011570159X361653250213114821

**Published:** 2025-02-24

**Authors:** Alexander Pavlovich Kalinin, Ekaterina Sergeevna Zubkova, Mikhail Yuryevich Menshikov, Yelena Victorovna Parfyonova

**Affiliations:** 1 National Medical Research Centre of Cardiology Named after Academician E.I. Chazov, Ministry of Health of the Russian Federation, Moscow 121552, Russia;; 2 Department of Biochemistry and Regenerative Biomedicine, Faculty of Fundamental Medicine, Lomonosov Moscow State University, Moscow 119991, Russia

**Keywords:** Integrated stress response, Alzheimer's disease, Parkinson's disease, amyotrophic lateral sclerosis, eIF2, ATF4, prion, traumatic brain injury

## Abstract

The dysfunction of different cells lies in the pathogenesis of neurological diseases and is usually associated with cellular stress. Various stressors trigger the integrated stress response (ISR) signaling, whose highly conserved mechanism is primarily aimed at protecting a stress-exposed cell to cope as safely as possible with such stressful conditions. On the contrary, if a cell is unable to cope with excessive stress, the ISR can induce apoptosis. The ISR mechanism, whose main stage is the inhibition of translation machinery in favor of the synthesis of specific proteins, including the transcription factors ATF3, ATF4, CEBPA, and CEBPB, which function only as dimers and determine the uniqueness of the ISR response in each individual case, thus ensures different outcomes of the ISR. Inhibition of global protein synthesis is achieved through phosphorylation of eIF2α by PERK, HRI, PKR, or GCN2. To date, a number of compounds have been developed that modulate the ISR, including activators and inhibitors of the abovementioned ISR kinases as well as modulators of p-eIF2α dephosphorylation. They target different ISR stages, allowing a broad ISR modulation strategy. At the same time, there are no drugs that are both exceptionally safe and effective for the treatment of several neurological diseases, so there is an urgent need for new approaches to the treatment of these disorders. In this review, we represent ISR signaling as an important participant in the pathogenesis of neurological diseases. We also describe how various ISR modulators may become a part of future therapies for these diseases.

## 1 INTRODUCTION

Neurological diseases represent a significant challenge to global health systems. As of 2021, an estimated 3.4 billion people are affected by these diseases, with 11.1 million deaths recorded [1]. These data highlight the persistent and substantial burden that neurological diseases place on global healthcare systems. Despite the plethora of studies conducted in recent decades aimed at developing novel treatments for neurological diseases, current statistics reveal an urgent need for new, safe, and cost-effective therapies [1, 2]. To address this need, the World Health Organization has established an intersectoral global action plan on epilepsy and other neurological disorders for the period 2022-2031 [3]. This plan includes the directive: “Strengthen national institutional capacity for research and innovation, such as for the development of new drugs for neurological disorders, including for children, by improving research infrastructure, equipment, and supplies” [3]. This initiative reinforces the necessity of introducing new pharmaceutical agents into the therapeutic armamentarium for the treatment of various neurological conditions that go beyond symptomatic management to address underlying disease mechanisms. One promising area of research is the targeting of the integrated stress response (ISR).

The ISR is a highly conserved cellular mechanism that enables cells to adapt to a variety of stressors, including amino acid starvation, viral RNA presence, mitochondrial dysfunction, and oxidative stress [4]. Upon activation, the ISR temporarily halts global protein synthesis while selectively permitting the translation of specific mRNAs, such as those encoding transcription factors that orchestrate the cellular stress response [4, 5]. These transcription factors, depending on their interaction partners, shape the cell's transcriptional response to promote adaptation or, under severe conditions, sensitize cells to apoptosis [6].

The role of ISR signaling has been widely studied in various pathological contexts, including cellular senescence, hematological malignancies, and ischemic and pulmonary diseases [7-10]. Given its central involvement in diverse disease pathways and the availability of targeted ISR modulators, these compounds hold potential for integration into clinical practice [11]. Recent research has increasingly implicated the dysregulation of the ISR in the pathogenesis of several neurological disorders, such as Alzheimer’s disease (AD), Parkinson’s disease (PD), Amyotrophic Lateral Sclerosis (ALS), and other neurodegenerative and cognitive conditions.

In the brain, the ISR plays a dual role: while transient activation can be neuroprotective by enhancing protein quality control and supporting cellular recovery, prolonged activation may lead to deleterious outcomes, including impaired synaptic plasticity, neuronal death, and protein aggregation. This duality makes the ISR a “double-edged sword” in brain health. Understanding the precise regulation of the ISR is therefore crucial, as both insufficient and excessive ISR activity can contribute to disease progression.

The aim of this review is to summarize the results of various studies on the use of ISR modulators in neurological disease models and to highlight how these strategies can be used to optimize ISR activity for therapeutic benefit. It also provides a comprehensive overview of a promising frontier in the treatment of neurological disorders. Fine-tuning ISR activity may offer novel avenues to mitigate neurodegenerative phenotypes and improve outcomes for patients suffering from these complex diseases.

## 2 MATERIALS AND METHODS

A systematic search of the literature was performed on September 12, 2024, in PubMed and Web of Science using the combination of terms “brain” or “neuron” or “neurological” with “ISRIB” or “GSK2606414” or “GSK2656157” or “trazodone” or “salubrinal” or “guanabenz” or “sephin1”. Titles and abstracts were screened by reviewers (AK and EZ) to exclude irrelevant articles. Exclusion criteria were defined as follows: reviews, conference abstracts, method/theory papers (without primary data), editorials, and articles written in a language other than English. Then, abstracts of the selected articles were screened to include “disease” or “disorder” by reviewers (AK and EZ), full text of the remaining articles was analyzed by reviewers (AK, EZ, and MM). The text of the review was originally written in English by non-native English-speaking reviewers (AK and EZ), so an AI-based tool, DeepL (deepl.com), was used to check the grammar of the written text. The final text was triple-checked by reviewers (AK, EZ, and MM) for any inaccuracies and compliance with genuine meaning.

## 3 MOLECULAR MECHANISM OF THE INTEGRATED STRESS RESPONSE AND ITS IMPLICATION IN NEUROLOGICAL DISEASES

The ISR plays a crucial role in maintaining cellular homeostasis under various stress conditions and has been increasingly recognized as a pivotal factor in neurological disorders.

A key function of this pathway is to modulate general protein synthesis in the cell, reducing global protein translation while promoting the selective translation of specific mRNAs that support cell survival under stress conditions. This switch is primarily mediated by the phosphorylation of the α-subunit of the eukaryotic translation initiation factor 2 (eIF2α, also known as eIF2S1) at the Ser51 residue. This phosphorylation event leads to a repression of overall protein synthesis while stimulating the translation of specific mRNAs that typically contain upstream open reading frames (uORFs) in their 5' untranslated regions (UTRs). This mechanism triggers a sequence of events necessary to maintain cell function during stress and recovery [4].

A distinctive feature of the ISR is the upregulation of the expression of the basic leucine zipper (bZIP) transcription factor, Activating Transcription Factor 4 (ATF4) [12]. Mammalian ATF4 mRNA is regulated by two upstream open reading frames within its 5' UTR. Under normal conditions, translation is repressed by these uORFs; however, when eIF2α is phosphorylated, translation initiates at the coding sequence for ATF4, leading to its increased expression [13].

Under normal, non-stressful conditions, eIF2α forms a heterotrimeric eIF2 complex together with eIF2β and eIF2γ [14]. This eIF2 complex then associates with GTP and initiator methionyl-tRNA (Met-tRNAi) to form a ternary complex (eIF2-GTP-Met-tRNAi). This ternary complex (TC) is crucial for the initiation of protein synthesis, as it binds to the 40S ribosomal subunit to form the 43S pre-initiation complex, initiating ribosomal scanning along the mRNA to locate the start codon. During translation initiation, guanosine triphosphate (GTP) within the TC is hydrolyzed to guanosine diphosphate (GDP), leading to the release of the Met-tRNAi into the ribosome [4].

Once the translation of the first upstream open reading frame is complete, the 40S subunit can reassociate with the available TC to reinitiate translation at the next uORF. This process is regulated by the guanine nucleotide exchange factor eIF2B, which catalyzes the exchange of GDP for GTP on eIF2, thereby regenerating the active eIF2-GTP complex necessary for maintaining high TC levels [4].

Under stress conditions, phosphorylation of eIF2α at Ser51 occurs, leading to the sequestration of eIF2B in an inactive complex with phosphorylated eIF2-GDP (p-eIF2-GDP) [15]. The inhibition of eIF2B prevents the recycling of eIF2-GDP to eIF2-GTP, thereby reducing the overall concentration of active ternary complexes. As a result, the 43S pre-initiation complex forms less efficiently, which decreases general protein synthesis [4].

eIF2α is phosphorylated in response to various stress conditions, including endoplasmic reticulum stress (ER-stress), oxidative stress, nutrient deficiency, proteotoxic shock, and viral infection. Four protein kinases are known to phosphorylate eIF2α under different types of stress [16]:

- Protein kinase R (PKR) is activated by double-stranded RNA generated during viral infection; Dysregulated PKR activity is also associated with memory impairments and seizures, highlighting its significance in various neurodevelopmental and neurodegenerative conditions [17]. PKR activation in these contexts may lead to aberrant translational control and increased neuroinflammation, exacerbating disease pathology.
- PKR-like Endoplasmic Reticulum Kinase (PERK) is primarily activated during ER stress, which occurs when unfolded or misfolded proteins accumulate in the ER lumen. This activation is a critical component of the unfolded protein response (UPR), a cellular mechanism aimed at restoring ER homeostasis. Unfolded proteins are a hallmark of several neurodegenerative diseases such as Alzheimer's disease, Huntington's disease, and amyotrophic lateral sclerosis. In these diseases, sustained PERK activation can contribute to synaptic dysfunction and neuronal loss, thereby worsening cognitive and motor impairments [18].
- The heme-regulated inhibitor protein kinase (HRI) is activated in erythrocyte precursors by iron deficiency, arsenite, heat shock, or osmotic stress. HRI kinase is an essential sensor of mitochondrial dysfunction and a key regulator of mitophagy, a cellular process aimed at the autophagic degradation of excess or damaged mitochondria [19]. HRI missense variants can cause a broad phenotypic spectrum, including variable cognitive impairment, developmental delay, hypertonia, white matter changes, hypotonia, ataxia, and involuntary movements [20]. Given that, the kinase is essential for the healthy development and functioning of an organism.
- A general control nonderepressible-2 protein kinase (GCN2) is activated in response to amino acid deficiency, UV irradiation, proteasome inhibition, and ribosome collisions. GCN2 plays a central role in amino acid metabolism and the integrated stress response by promoting adaptation to nutrient deprivation. Dysregulated GCN2 activity has been observed in several neurodegenerative disorders, including Alzheimer's disease. While deletion of GCN2 has been shown to alter memory formation and synaptic plasticity, its chronic activation may contribute to neurodegeneration by disrupting protein synthesis and promoting aberrant stress responses [21].

In addition to their conserved catalytic domains, these kinases possess unique regulatory domains that enable them to sense specific stress signals, leading to dimerization and trans-autophosphorylation. This activation mechanism ensures precise and context-specific phosphorylation of eIF2α, thereby modulating the integrated stress response according to the nature and intensity of the stress encountered [5].

A characteristic feature of the ISR is its reversibility. The suppression of the ISR and the restoration of general protein synthesis are primarily mediated by the dephosphorylation of the eIF2α subunit. This dephosphorylation is carried out by two heterodimeric phosphatases composed of the catalytic subunit protein phosphatase 1 (PP1) and one of two non-catalytic regulatory subunits: PPP1R15A (GADD34, growth arrest and DNA damage-inducible gene 34) or PPP1R15B (CReP, constitutive repressor of eIF2α phosphorylation) [22].

The regulatory subunit GADD34 is induced as part of a feedback loop that helps terminate the stress response once homeostasis is restored. By recruiting PP1 to dephosphorylate eIF2α, GADD34 promotes the resumption of protein synthesis following the cessation of stress. This feedback mechanism is critical for cellular recovery and adaptation; however, under chronic or unresolved stress conditions, premature translation reactivation can lead to proteotoxic stress, exacerbate protein misfolding, and trigger apoptosis [4].

ATF4, a key transcription factor expressed during ISR activation, plays a central role in coordinating the cellular response to diverse stressors [5]. ATF4 was initially characterized as a transcriptional repressor of cyclic AMP response elements (CRE) [23]. The multifaceted effects of ATF4 on gene expression are largely attributed to its ability to form heterodimers with a wide range of other transcription factors, such as bZIP transcription factors: JUN, FOS, and FRA1, to bind CRE sites [5]. Additionally, ATF4 can form heterodimers with CCAAT/enhancer-binding protein gamma (C/EBPG), enabling it to bind C/EBP-ATF response elements [24]. Another prominent interaction partner of ATF4 is C/EBP homologous protein (CHOP), which, under certain stress conditions, mediates pro-apoptotic effects [25]. The ATF4 interactome includes other protein factors such as C/EBPB, C/EBPE, C/EBPA, NRF2, NFE2L1, CREBZF, MAF, and some other transcription factors [25-29].

The specific dimerization partners of ATF4 significantly influence the selection of target DNA sequences, thereby modulating the expression of genes involved in amino acid metabolism, redox regulation, and apoptotic signaling [5]. This dynamic regulation allows ATF4 to adapt the cellular response according to the type and severity of the stress encountered, contributing to either cell survival or programmed cell death [5].

In Parkinson's disease, ATF4 has emerged as a key regulator of dopaminergic neuronal death. Exposure to PD-associated neurotoxins (*e.g*., 6-hydroxydopamine (6-OHDA) and 1-methyl-4-phenylpyridinium), as well as α-synuclein aggregation, induces prolonged ATF4 activation. This sustained activation promotes the expression of pro-apoptotic genes such as Chop, Trb3, and Puma, leading to increased neuronal apoptosis [30].

Similarly, in AD, ATF4 has been shown to play a role in the regulation of mitochondrial stress responses. O-GlcNAcylation, a post-translational modification involving the addition of O-linked N-acetylglucosamine, can modulate ATF4 activity and its downstream targets. In AD, dysregulation of O-GlcNAcylation disrupts ATF4-mediated signaling pathways, contributing to impaired mitochondrial function and increased neuronal vulnerability [31]. This finding suggests that modulating ATF4 and related ISR pathways could represent a potential therapeutic strategy in neurodegenerative diseases.

The repertoire of genes regulated by ATF4 during the ISR is extensive, encompassing those involved in amino acid metabolism, antioxidant responses, and apoptosis. Through its interactions with diverse transcription factors, ATF4 can adapt the transcriptional response to specific stressors, ultimately influencing cell viability, differentiation, and metabolism. This versatility allows ATF4 to act as a critical determinant of cell fate, modulating survival or apoptotic pathways depending on the nature and intensity of the stress signal [5].

Dysregulated chronic phosphorylation of eIF2α leads to global inhibition of protein synthesis, affecting synaptic plasticity and long-term potentiation that are critical for learning and memory. Such dysregulation has been implicated in several neurodegenerative conditions, contributing to neuronal death, memory impairments, and protein aggregation [32, 33].

Experimental studies have demonstrated that mice heterozygous for an alanine mutation at the Ser51 residue of eIF2α (which prevents its phosphorylation) exhibited enhanced long-term memory and improved memory consolidation. In contrast, pharmacological induction of eIF2α phosphorylation had the opposite effect, impairing memory and synaptic function [34]. Similarly, pharmacological downregulation of ATF4 in mice resulted in enhanced synaptic plasticity and long-term memory [35]. Pathological features of neurodegenerative diseases, such as amyloid and tau aggregation, repeat-associated non-AUG translation, and neuroinflammation, have been linked to the activation or dysregulation of ISR kinases. Altering the activity of ISR kinases, such as PERK or GCN2, modulates these distinct pathological pathways and influences disease progression [33].

ISR activity is known to modulate neuroimmune interactions and influence cognitive functions, which are crucial in stress-related disorders, such as those triggered by trauma or chronic sleep deficiency. Chronic sleep loss, for instance, can activate ISR pathways, thereby exacerbating neuronal damage and cognitive decline [36, 37].

Given the multifaceted roles of the ISR in the brain, a comprehensive understanding of its dynamics is crucial for developing effective therapeutic interventions for neurological disorders. Several recent studies have shown promising results in pharmacologically modulating the ISR to alleviate neurodegenerative phenotypes and restore synaptic function. Such strategies include targeting specific ISR kinases or downstream effectors to fine-tune the balance between adaptive and maladaptive stress responses.

With the advent of numerous specific inhibitors and activators, researchers now have the tools to effectively modulate nearly all steps of the ISR signaling pathway, making precise targeting of ISR kinases and downstream effectors highly accessible. These modulators can be categorized into three groups depending on their point of application within the ISR machinery. The first group comprises modulators of ISR kinases: activators (histidinol, halofuginone) [38, 39] and inhibitors (indirubin-3-monoxime, SP600125) [40] of GCN2 kinase; activators (BTdCPU, cHAUs) [41, 42] and inhibitor (aminopyrazolindane) [43] of HRI kinase; activators (BEPP, CE7-93) [44, 45] and inhibitors (C16, C51) [46] of PKR kinase; activators (CCT020312, MK-28) [47, 48] and inhibitors (GSK2606414, GSK2656157) [49, 50] of PERK kinase. The second group comprises activators of eIF2B (ISRIB, 2BAct) [51, 52] and suppressors of the inhibitory effect of eIF2α on eIF2B (trazodone, dibenzoylmethane) [53]. The third group comprises inhibitors of p-eIF2α dephosphorylation - specifically, inhibitors of GADD34 (salubrinal, guanabenz, sephin1) [54-56] and CReP (salubrinal, raphin1) [54, 57] which are regulatory subunits of the PP1 complex.

A simplified schematic representation of the ISR pathway, along with the key pharmacological modulators highlighted in the review, is shown in Fig. (**1**).

Some of these compounds, such as FDA-approved drugs trazodone and guanabenz, were originally developed for other therapeutic purposes and were only later found to modulate ISR signaling. In contrast, others, like GSK2606414, GSK2656157, and sephin1, were specifically designed for their high selectivity in targeting ISR components. To provide a comprehensive understanding of ISR modulation in neurological diseases, this review focuses on a representative selection of ISR modulators: widely used ISR modulators (salubrinal and ISRIB), highly selective ISR inhibitors (GSK2606414, GSK2656157), and FDA-approved drugs with off-target effects on the ISR (trazodone, sephin1 and guanabenz). This approach allows us to evaluate both general and specific ISR modulators, highlighting their therapeutic potential and mechanisms of action in neurological diseases.

## 4 MODULATORS OF ISR AS THERAPEUTICS IN NEUROLOGICAL DISEASES

### 4.1 ISR Inhibitors in Neurological Diseases

#### 4.1.1 ISRIB

Integrated Stress Response Inhibitor (ISRIB; (2-(4-chlorophenoxy)-N-[[1, 4]-4-[2-(4-chlorophenoxy) acetamido] cyclohexyl] acetamide) has gained considerable attention in recent years due to its unique ability to enhances memory and modulate ISR pathway [51]. This effect is mediated through ISRIB’s ability to promote more favorable conformational changes in eIF2B, which stabilizes its interaction with eIF2 [51]. This modulation increases the availability of guanine nucleotide exchange factor activity of eIF2B within the cell, ensuring that the residual amount not engaged with phospho-eIF2α is sufficient to maintain normal levels of the eIF2•GTP•Met-tRNAi ternary complex to support normal cellular function [51]. Notably, ISRIB has the ability to cross the blood-brain barrier with ease, rendering it a promising candidate for the treatment of brain conditions [51, 58].

ISRIB was initially discovered for its remarkable ability to enhance memory. The Sidrauski research group conducted a targeted screen for inhibitors of PERK signaling [18] and selected ISRIB from a pool of 28 candidate chemicals [51]. ISRIB has been shown to enhance long-term memory formation and facilitate both hippocampus-dependent spatial learning and hippocampus-dependent contextual fear conditioning in murine models [51]. ISRIB treatment in aged mice effectively counteracted age-related declines in spatial learning and memory, enhanced cognitive performance, and rejuvenated neuronal function to levels comparable to those observed in young mice, with effects persisting weeks after treatment onset [59]. These outcomes were partially attributed to ISRIB’s ability to modulate intrinsic excitability and reduce neuronal hyperpolarization following high-frequency firing [59]. Moreover, ISRIB treatment significantly increased the number of dendritic spines and modulated immune profiles, particularly by attenuating interferon and T cell-mediated responses [59]. Furthermore, the authors suggest that elevated ISR signaling activation in the brain may serve as a marker of neurodegenerative malfunction [59]. Given its ability to enhance cognitive function, counteract age-related declines, and modulate both neuronal and immune profiles, ISRIB warrants further investigation as a potential therapeutic agent for a range of neurological disorders.

##### 4.1.1.1 Alzheimer's Disease

Clinical evidence suggests that the age-associated progressive cognitive decline observed in patients with Alzheimer's disease is linked to the extent of tau pathology- specifically, the degree and nature of tau phosphorylation, as well as the accumulation of β-amyloid in senile plaques [60, 61]. Zhang and colleagues showed that low-frequency stimulation (LFS) leads to the elevation of tau phosphorylation through mechanisms involving N-methyl-D-aspartate receptors (NMDARs) and metabotropic glutamate receptor 5 (mGluR5) in aged rats. Notably, this effect was not observed in either ISRIB-treated aged rats or in young, 2-3-month-old rats [60]. Regrettably, the authors did not elucidate the precise mechanism underlying ISRIB’s protection against LFS-induced tau phosphorylation [60]. Moreover, the study did not explore potential changes in ISR effector proteins that could provide further insight into ISRIB’s protective effects [60].

Elevated levels of tyrosine have been shown to worsen cognitive function in elderly individuals and patients with AD and to promote axonal degeneration and demyelination in patients with tyrosinemia [62]. These detrimental effects are associated with reduced levels of tyrosyl-tRNA synthetase (YARS1) and phenylalanyl-tRNA synthetase beta (FARSB) in the affected brains [62]. Notably, tyrosine has been shown to inhibit the activation of poly-ADP-ribose polymerase 1 (PARP1), which is an essential enzyme involved in neuronal DNA repair [63]. Treatment with low doses of ISRIB (5-50 nM) has been shown to counteract these changes by increasing the protein levels of YARS1 and FARSB, thereby providing neuroprotective benefits [62]. Conversely, higher doses of ISRIB (250-500 nM) result in a reduction in YARS1 levels [62].

ISRIB’s protective effect against neuronal shrinkage and degeneration was demonstrated by Goswami and colleagues in a rat model of experimentally induced Alzheimer’s disease. They showed that ISRIB treatment alleviated Aβ [1-42]-induced cognitive impairment, reduced the pNF-κB/NF-κB ratio, and downregulated pro-inflammatory proteins such as interleukin-1 beta (IL-1β), cyclooxygenase-2, and tumor necrosis factor-alpha (TNF-α), while simultaneously upregulating anti-inflammatory cytokines IL-4 and IL-10 [64]. ISRIB also decreased the levels of ISR pathway molecules, including CHOP, ATF4, and GADD34 [64]. However, the study did not establish a clear molecular link between ISRIB’s effects and symptom relief, nor did it assess changes in PERK or eIF2α phosphorylation to confirm the modulation of ER stress [64]. In turn, Hosoi and colleagues demonstrated that ISRIB protected against Aβ-induced neuronal cell death in PC12 cells without altering Aβ production [61]. Hu and colleagues further elucidated ISRIB’s protective mechanism, showing that the injection of soluble Aβ [1-42] in their model led to long-term depression and activation of ISR signaling, which restricted protein synthesis [65]. ISRIB counteracted these effects by restoring protein synthesis and preventing Aβ [1-42]-induced LTD, ultimately improving learning and memory [65]. This finding is consistent with observations by Oliveira and colleagues, who reported that brain tissues from AD patients exhibit increased levels of phosphorylated eIF2α and diminished eIF2B subunit concentrations [66]. They also demonstrated that ISRIB can reverse p-eIF2α-mediated deficits in long-term memory in both salubrinal-treated mice and AD mouse models and enhance synaptic function and cognitive performance in AD mice [66]. Collectively, these studies suggest that ISRIB has a beneficial effect on AD pathogenesis by reducing neuroinflammation and partially mitigating memory decline. However, further research is needed to fully understand the impact of ISRIB-mediated modulation of the ISR pathway on disease progression.

##### 4.1.1.2 Amyotrophic Lateral Sclerosis (ALS), Prion Disease, Noise-induced Cochlear Synaptopathy and Down Syndrome

A loss or reduction in the synthesis of vital proteins by neuronal cells is a hallmark of neurodegeneration, and many neurological diseases are characterized by elevated levels of phosphorylated eIF2α (p-eIF2α). Consequently, ISRIB has been widely used to restore protein translation in various brain conditions [33]. For example, ISRIB partially rescues protein synthesis rates in prion-affected mice without causing the pancreatic toxicity observed with the PERK inhibitor GSK2606414 [58]. Additionally, ISRIB treatment downregulates ATF4, prevents neuronal loss in the hippocampus, and reduces typical prion-induced spongiform pathology [58].

In a rodent model of amyotrophic lateral sclerosis (ALS), ISRIB alleviated ER stress and enhanced the survival of G93A SOD1-expressing neurons, in contrast to GSK2606414 treatment [67]. However, ISRIB did not improve the survival of E46K α-synuclein-expressing neurons, highlighting the specificity of its mechanism of action [67]. Interestingly, ISRIB reduced ATF4 levels as effectively in microtubule-associated protein 2-positive (MAP2+) neurons as GSK2606414 did in non-neuronal MAP2- cells [67].

ISRIB rescued the impairment in long-term memory and late long-term potentiation (L-LTP) in the Ts65Dn murine model of Down syndrome, although it did not further enhance L-LTP or long-term fear memory in WT mice [68].

In a murine model of noise-induced cochlear synaptopathy, ISRIB significantly reduced noise-induced threshold elevation and wave-I amplitude reduction on both day 1 and day 21 post-noise exposure, thereby protecting against noise-induced synapse loss [69]. Interestingly, the study suggested that the effects of ISRIB might be sex-dependent, as male animals responded more favorably to the treatment compared to females [69].

The collective evidence indicates that ISR activation occurs in neurons in prion diseases, noise-induced cochlear synaptopathy, ALS, and Down syndrome. The administration of ISRIB has been shown to relieve symptoms of these diseases by reducing the cell apoptosis rate and enhancing memory function, in particular through the downregulation of the key ISR-related transcription factor ATF4.

##### 4.1.1.3 Neurological Injuries

In the acute phase, transient neuroinflammation is beneficial, stimulating an anti-inflammatory response to the damage after SCI, TBI, and other neurological injuries [70-72]. In contrast, chronic neuroinflammation is clearly deleterious, causing disruption to the blood-brain barrier, oxidative stress, excitotoxicity, and mitochondrial dysfunction in neurons and surrounding cells [70-72]. As previously noted, activation of the ISR pathway has been observed in a variety of neurological diseases [33].

Neurons in the vicinity of the epicenter of the spinal cord injury (SCI) exhibit elevated levels of p-eIF2α [73]. Administration of ISRIB after SCI restores protein synthesis, reducing neuron loss in the anterior horn, minimizing tissue cavity formation, and improving the hind limb's locomotor function [73]. Similarly, in a mouse model of traumatic brain injury (TBI), ISRIB treatment reversed cortical spine dynamics changes and restored working memory following closed-head injury (CHI) [74]. Remarkably, these restorative effects persisted for several weeks after the ISRIB treatment ended, lasting well beyond the time required for the drug to be fully cleared from the body [74].

ISRIB has been shown to effectively reverse deficits in spatial learning and memory consolidation caused by TBI [75]. Furthermore, It also restores working and episodic memory deficits in a concussive injury model and reverses TBI-induced impairments in hippocampal long-term potentiation (LTP) in C57B6/J mice [75]. *In vitro* ISRIB suppressed lipopolysaccharide(LPS)-induced M1 polarization of BV-2 microglial cell line and promote their switch towards the M2 phenotype, resulting in a marked reduction of TNF-α and IL-6 secretion, along with a significant increase in IL-10 and TGF-β production [76]. *In vivo* ISRIB administration alleviated microglia infiltration and improved both neurological and motor function in a rat model of surgical brain injury (SBI) [76].

The characteristic features of a murine model of postoperative cognitive dysfunction (POCD) induced by tibial fracture surgery include cognitive decline, oxidative stress damage, and ISR activation in the hippocampus [77]. ISRIB treatment mitigated cognitive dysfunction in POCD mice, reduced oxidative stress, and suppressed ISR activation, as evidenced by a lower p-eIF2α/eIF2α ratio and decreased levels of ATF4, CHOP, and GADD34 proteins [77].

In another experimental setup, ISRIB treatment alleviated high glucose (50 mM)-induced neuronal injury by decreasing the levels of p-eIF2α, CHOP, and ATF4, which were elevated during the injury [78]. Notably, this study showed that the effects of resveratrol are comparable to those of ISRIB [78].

These findings indicate that the ISRIB can reduce the defects induced by POCD, SCI, and TBI, enhancing memory function and alleviating neuroinflammation. This is accompanied by a clear suppression of the ISR pathway, suggesting that the ISR signaling is capable of setting up a proinflammatory secretory phenotype in neuroinflammation.

##### 4.1.1.4 Fragile X Syndrome (FXS)

Fragile X syndrome (FXS) represents the most widespread inherited form of intellectual disability and autism spectrum disorder, with an estimated prevalence of 1 in 5,000 in males and 1 in 4,000 to 1 in 8,000 in females [79]. The etiology of FXS is attributed to the loss of fragile X messenger ribonucleoprotein (FMRP), an RNA-binding protein that regulates the translation of approximately 4% of all brain transcripts, including PSD-95, GluA1, GluA2, ARC, and MAP1B. The absence of FMRP leads to dysregulated synaptic protein levels, with increased PSD-95 stabilizing dendritic spines but reducing glutamate receptor accumulation, ultimately resulting in the formation of dense, immature dendritic spines. ISRIB treatment has been shown to stabilize cortical spike dynamics and improve social recognition. It also increases GluA1 levels at the postsynaptic terminal, thereby normalizing glutamate receptor-mediated synapse maturation. However, ISRIB does not restore global protein synthesis in FMR1 knockout (KO) mice [80]. The current research precludes a thorough assessment of the role of the ISR pathway in the pathophysiology of fragile X syndrome.

In summary, ISRIB is a promising candidate for the treatment of various neurological diseases. However, as previously discussed, the opposing compound, salubrinal, an activator of ISR signaling, also shows potential for treating a broad range of brain disorders. Additionally, evidence suggests that ISRIB may not always provide neuroprotection. For example, ISRIB failed to rescue spatial learning and memory deficits in transgenic J20 mice overexpressing human amyloid-beta precursor protein (hAPP), and no differences in ATF4 protein levels were observed between healthy non-transgenic and hAPP-J20 mouse brains [81].

The PS19 transgenic mice, a model for tauopathy, did not show ER stress-related increases in ATF4 or CHOP but exhibited spatial learning and memory deficits. While ISRIB modestly restored spatial memory acquisition in these mice, its administration was associated with worsened p-tau neuropathology [82]. ISRIB was also ineffective in restoring locomotor hyperactivity, fear-based learning, memory deficits, impaired spatial working memory, and recognition memory in amyloidosis-modeling APPSwe transgenic mice [82].

Additionally, in a murine model of neuropathy with prohibitin 1 specifically knocked out in Schwann cells, ISRIB administration led to increased axon demyelination, myelin degradation (myelinophagy), and reduced performance in the rotarod test [83]. Moreover, ISRIB failed to reverse the severe translational arrest observed in an *in vitro* model of amyotrophic lateral sclerosis and frontotemporal dementia (ALS/FTD) [84].

In conclusion, although ISRIB has demonstrated significant potential in alleviating various cognitive and behavioral deficits associated with neurodegenerative and neuroinflammatory conditions. Its ability to restore protein synthesis, reduce ER stress, and modulate synaptic function highlights its therapeutic value across a range of neurological diseases, including Alzheimer’s disease, traumatic brain injury, and Fragile X syndrome. However, ISRIB’s efficacy is not universal, as evidenced by its lack of neuroprotection in specific models, such as amyloidosis and tauopathy, where it even worsened certain pathological features. These findings underscore the need for a deeper understanding of ISRIB’s mechanism of action and its context-specific effects before its therapeutic application can be fully realized. Further research should focus on identifying the precise conditions under which ISRIB can provide optimal benefits while minimizing potential adverse outcomes.

#### 4.1.2 GSK2606414 and GSK2656157

Axten and colleagues developed a potent and selective protein kinase R (PKR)-like endoplasmic reticulum kinase (PERK) inhibitor 7-Methyl-5-(1-{[3-(trifluoromethyl)phenyl]- acetyl}-2,3-dihydro-1H-indol-5-yl)-7H-pyrrolo [2,3-d]pyri-midin-4-amine (GSK2606414), that could penetrate blood-brain barrier [49, 85]. Subsequently, they introduced GSK2656157 (1-(5-(4-Amino-7-methyl-7H-pyrrolo [2,3-d]-pyrimidin-5-yl)-4-fluoroindolin-1-yl)-2-(6-methylpyridin-2-yl) ethenone), an optimized derivative with improved physical properties and pharmacokinetics [50]. Both compounds were designed to selectively inhibit PERK, a key mediator of UPR and ER stress signaling pathways [49, 50]. Elevated levels of phosphorylated PERK (p-PERK) have been observed in various neurological disorders, highlighting the potential therapeutic applications of PERK inhibitors in treating brain conditions [86].

ER stress models induced by tunicamycin or thapsigargin are frequently applied to evaluate the impact of specific proteins or compounds on UPR and ER stress signaling pathways [87]. Smith *et al*. investigate how the activation of the UPR in astrocytes leads to a reactive state that adversely affects neighboring neurons in a murine model. The study demonstrates that UPR activation in astrocytes by thapsigargin, specifically through the PERK-p-eIF2α-ATF4 pathway, induces a distinct reactive phenotype characterized by the upregulation of Cxcl10, Lcn2, and Vim. Inhibiting PERK with GSK2606414 prevents these changes [88]. Furthermore, PERK inhibition restored the synaptogenic properties of astrocyte-conditioned media (ACM), with ACM derived from GSK2606414-treated, thapsigargin-stressed astrocytes significantly increasing synapse number by 1.47-fold [88]. These findings suggest that targeting the PERK pathway in astrocytes may offer therapeutic potential for neurodegenerative diseases.

##### 4.1.2.1 Diabetes Mellitus and Ischemic Processes, Injuries and Hemorrhages

ER-stress, particularly PERK and ISR signaling pathways, has been established as a pivotal player in the pathogenesis of diabetes mellitus and ischemic processes [89, 90]. Indeed, GSK2606414 has been demonstrated to alleviate a number of pathological features in a rodent model of cerebral ischemia [91]. These include neurological deficits, brain infarct volume, cognitive and recognition memory decline, muscle and locomotor weakness, oxidative stress, and pro-inflammatory cytokines [91]. Additionally, GSK2606414 has been shown to mitigate the mRNA expression of a number of proteins, including GFAP, GAP43, Bax, Bad, Bcl2, PERK, ATF4, and CHOP, which are induced by cerebral ischemia [91]. Furthermore, GSK2606414 has been observed to enhance synaptic protein expression [91]. Following a two-week exposure of C57BL/6J mice to intermittent hypoxia (IH), Xu and colleagues observed elevated levels of ISR effector proteins p-PERK, p-elf2α, and ATF4 in the hippocampus, but not ATF6 and p-IRE1 [92]. In turn, treatment with GSK2606414 led to a notable reduction in IH-related symptoms: restoration of ER morphology, rescue of memory impairment, and alleviation of mitochondrial-dependent apoptosis through a reduction in the expression of pro-apoptotic proteins CHOP, Bim, and cleaved caspase-3 [92].

To model diabetic neuropathy, Gundu *et al*. treated neuroblastoma N2A cells with high glucose and observed increased levels of p-eIF2α/eIF2α, ATF4, CHOP, and GRP78, a marker of ER stress. GSK2606414 reduced these protein levels, alleviated neuronal apoptosis by upregulating Bcl-2 and downregulating Bax and Caspase-3, and improved mitochondrial function by increasing TFAM, NRF1, ATP synthase, and mitochondrial complexes 1 and 2 [93]. These findings suggest that the ISR signaling pathway is an essential component of the pathogenesis of diabetic neuropathy and brain ischemic processes, with GSK2606414 showing potential to reduce apoptosis and enhance memory function.

Although ER stress plays a beneficial role in wound regeneration, its persistent activation has been implicated in the pathogenesis of various brain injuries and hemorrhages [94]. In primary rat cortical neurons, intracerebral hemorrhage (ICH) was shown to be associated with increased levels of p-eIF2α/eIF2α, ATF4, CHOP, and cleaved caspase-12. GSK2606414 reversed these elevations both *in vivo* and *in vitro*, preventing ICH-induced neuronal apoptosis [95]. In contrast, salubrinal, by impeding eIF2α dephosphorylation, increased p-eIF2α levels and activated the downstream PERK pathway, leading to neuronal apoptosis and necrosis following ICH *in vitro* and *in vivo* [95]. Similarly, the PERK-ATF4-CHOP pathway appears to play a key role in stress-induced hypothalamic neuronal injury, as shown in a rat model by Yi *et al*. GSK2606414 application significantly decreased ATF4 and CHOP protein levels and reduced cell damage, as confirmed by thionine staining [34].

Wu and colleagues demonstrated that rats subjected to SBI showed elevated levels of ISR effectors, including p-PERK, p-eIF2α, ATF4, and caspase-3, which were reversed by GSK2606414. This treatment improved neurological and behavioral scores, inhibited apoptosis, and promoted neuronal survival. Similarly, in a subarachnoid hemorrhage model, GSK2606414 reduced brain edema, enhanced neuronal survival, and mitigated neurological deficits 72 hours post-injury. It also decreased levels of ISR effectors (p-PERK, p-eIF2α, ATF4) and increased the Bcl-2/Bax ratio [96].

Collectively, these findings suggest that treatment with GSK2606414 in a range of brain injuries and hemorrhages results in a notable suppression of the ISR pathway, accompanied by a reduction in neuronal apoptosis and a restoration of protein synthesis.

##### 4.1.2.2 Alzheimer's Disease

The initial stages of AD are characterized by mild ER stress, which activates the UPR to promote cellular resilience. However, in advanced disease stages, sustained ER stress drives a maladaptive UPR that exacerbates disease progression [97].

In the rTg(tauP301L+)4510 murine model, Radford *et al*. showed that elevated mutant tau protein expression activated the PERK branch of the UPR, resulting in increased p-eIF2α/eIF2α and ATF4 levels, along with sustained translational repression. GSK2606414 administration reduced tau phosphorylation, mitigated brain atrophy, and improved clinical signs by decreasing the p-eIF2α/eIF2α ratio and restoring global translation rates [98].

Using the same tauopathy mouse model, Koren *et al*. demonstrated that GSK2606414 rescued brain atrophy and functional deficits without affecting tau hyperphosphorylation. They also proposed that functional deficits in early-stage tauopathy are independent of PERK/UPR activation, as p-PERK and ATF4 levels were unaltered in rTg4510 mice [99]. The authors also demonstrated that the beneficial effect of GSK2606414 in rTg4510 mice was PERK-independent. Proteomic analysis revealed that GSK2606414 treatment restored several proteins implicated in neuronal function and synaptic stability, including NUCB1, SNCA, FKBP1A, PKM, YWHAZ, HSPE1, and HOMER1 [99]. These findings indicate that GSK2606414 may exert neuroprotective effects through mechanisms beyond PERK inhibition.

##### 4.1.2.3 Amyotrophic Lateral Sclerosis

The pathologic mechanisms of ALS are known to be highly dependent on the ER-stress pathway since a hallmark of ALS is an abnormal accumulation of misfolded or aggregated proteins (mutant SOD1, TDP-43) in affected neurons and glia [100]. Notably, Kim *et al*. demonstrated that TDP-43 and ataxin-2 proteins are linked to ALS pathogenesis, particularly within stress granules, and that prolonged eIF2α phosphorylation exacerbates TDP-43 toxicity. These findings suggest that mitigating eIF2α phosphorylation with compounds like GSK2606414 could be a potential therapeutic strategy for ALS and related proteinopathies [101].

Hexanucleotide repeat expansions in the C9orf72 gene represent the most common cause of ALS and FTD [102]. Gao *et al*. demonstrated that GSK2606414 reduces poly(GA) accumulation and autophagy dysfunction in induced pluripotent stem cell-derived C9orf72 neuromuscular organoids. However, its effects on skeletal muscle (SKM) were dosage-dependent: high doses worsened SKM defects, possibly by reducing nicotinic acetylcholine receptors, whereas moderate doses enhanced SKM contraction under glutamine supplementation [102]. Similarly, Szebényi *et al*. observed in human ALS/FTD brain organoids that GSK2606414 mitigated astroglial autophagy dysfunction, poly(GA) accumulation, DNA damage, and nuclear pyknosis [103].

Taken together, these findings suggest that GSK2606414 alleviates ALS-related symptoms by reducing pathogenic protein aggregates and restoring cellular function. However, the compound’s potential adverse effects at high doses underscore the need for further studies to optimize its use in ALS models.

##### 4.1.2.4 Parkinson’s Disease

ER stress and UPR, primarily activated by αS protein aggregates, are key factors in Parkinson’s disease (PD) pathogenesis [104]. Mercado *et al*. demonstrated that brain tissues from PD patients and αSyn^A53T^Tg transgenic mice exhibited elevated levels of p-PERK, p-eIF2α, and ATF4, indicating ISR pathway activation [105]. Treatment with GSK2606414 enhanced dopaminergic neuron survival in the substantia nigra pars compacta, improved dopamine levels, and restored motor functions in αSynA53TTg mice [105]. At the molecular level, GSK2606414 reduced pro-apoptotic signals downstream of ATF4 and restored synaptic protein levels [105]. However, prolonged administration (21 days) of GSK2606414 caused pancreatic toxicity, including pancreatic acinar disintegration and reduced islet content compared to vehicle controls [105]. These findings highlight the therapeutic potential of targeting UPR and ISR pathways in PD but underscore the need for caution due to the risk of significant side effects, such as pancreatic damage [58].

##### 4.1.2.5 Huntington's Disease

Huntington's disease (HD) is characterized by the presence of mutant huntingtin toxic oligomers, which induce misfolded protein accumulation in the ER, triggering the UPR pathway. UPR activation, in turn, leads to an aberrant increase in the levels of GRP78, CHOP, p-PERK, and p-eIF2α [106]. Espina and colleagues demonstrated that GSK2606414 treatment ameliorated the loss of apical dendritic spine density in pyramidal CA1 neurons in HD-modelling R6/1 mice while reducing the number of spines in treated WT mice [106]. These findings implicate the GRP78/PERK axis in HD-related hippocampal synaptic alterations and cognitive deficits [106]. Notably, GSK2606414 prevented memory decline in R6/1 mice independently of eIF2α phosphorylation, suggesting an alternative mechanism of action. The study highlights the potential of GSK2606414 in alleviating HD-induced ER stress and ISR, offering a promising therapeutic approach for the disease [106].

##### 4.1.2.6 Marinesco-Sjögren Syndrome

Marinesco-Sjögren syndrome (MSS) is a rare, early-onset, autosomal recessive multisystem disorder with three main hallmarks: cerebellar ataxia, cataracts, and myopathy [107]. Most cases result from loss-of-function mutations in SIL1, which encodes a nucleotide exchange factor for the chaperone BiP, essential for proper protein folding in the ER. This dysfunction triggers ER stress and activation of the PERK/eIF2α pathway, as observed in Purkinje cells (PCs) of woozy mice, a model for MSS. During PC degeneration, CHOP expression is upregulated, further contributing to the disease pathology [107]. Grande *et al*. demonstrated that treatment with GSK2606414 in presymptomatic woozy mice delayed neurodegeneration and motor dysfunction, extending the asymptomatic phase. In symptomatic mice, GSK2606414 attenuated motor impairments and skeletal muscle pathology [107]. Oral administration of GSK2606414 from the fifth week of life significantly delayed the onset of ataxia, with treated mice performing better vehicle-treated controls in rotarod and beam walking tests and exhibiting milder tremors [107]. The authors propose that inhibiting PERK signaling at early stages of MSS could substantially alleviate symptoms and delay neurodegeneration [107]. Thus, targeting ER stress and ISR with GSK2606414 holds promise as a potential approach to reduce MSS-related symptoms and postpone disease progression.

##### 4.1.2.7 Traumatic Brain Injury

GSK2656157, a PERK inhibitor related to GSK2606414, has also been extensively studied in neurological diseases. While GSK2606414 was the first potent PERK inhibitor identified and is widely used in research, GSK2656157 was developed as a derivative with improved pharmacokinetic properties and enhanced selectivity for PERK. Sen *et al*. demonstrated that TBI in mice is accompanied by PERK-associated ER stress, which subsequently activates the STING-TBK1-IRF3 pathway, leading to the synthesis of IFNβ in neurons [108]. GSK2656157 effectively mitigated TBI-induced Th1 cell infiltration, IFNβ-associated microglial activation, and polarization to the M1 phenotype. It also reduced elevated levels of phospho-STING, phosphor-TBK1, and phosphor-IRF3 associated with TBI. Additionally, GSK2656157 alleviated anxiety, depressive-like behaviors, brain tissue loss, and white matter damage in TBI models [108].

In a separate study, PERK activation was shown to impair memory in a murine TBI model by inactivating CREB and downregulating PSD95 [109]. Elevated phosphorylation of CREB at S129 hindered its transcriptional activity, while PSD95 phosphorylation at T19 led to its downregulation after TBI [109]. GSK2656157 alleviated memory impairment and promoted neurite outgrowth by reducing CREB and PSD95 phosphorylation without affecting elevated eIF2α phosphorylation [109]. Additionally, Liu *et al*. demonstrated that GSK2656157 reduced ER stress-related apoptotic protein expression in neuronal cells following TBI in a dose-dependent manner. Furthermore, at 1 μmol/L, GSK2656157 reversed changes in PERK, ATF4, CHOP, and GRP78 expression, inhibited PERK signaling, reduced apoptosis, and attenuated ER expansion in Neuro-2a cells [110]. This effect was observed in a dose-dependent manner [110]. Furthermore, 1 μmol/l GSK2656157 was observed to effectively reverse the alterations in PERK, ATF4, CHOP, and GRP78 expression [110]. In summary, GSK2656157 is often used alongside GSK2606414 for ER stress suppression as a superior analogue. However, its application in neurological disease models remains limited, highlighting the need for further investigation into its therapeutic potential in brain conditions.

Nevertheless, the impact of PERK inhibitors on neurological disorders remains complex and context-dependent. For instance, GSK2606414 exacerbated the loss of newborn neurons after TBI and impaired contextual discrimination [111]. Zhu *et al*. demonstrated that PERK positively regulates Ca^2+^-dependent working memory and protein synthesis-dependent memory flexibility. In contrast, GSK2606414-treated mice showed deficits in working memory (spontaneous alternation Y-maze task) and fear extinction, mimicking deficits seen in forebrain-specific Perk knockout mice [112].

In an ICH rat model, PERK activation increased the p-eIF2α/eIF2α ratio and ATF4 levels, reaching a peak at 12 hours following the onset of ICH [113]. PERK activation proved beneficial by improving neurological behavior, reducing brain edema, and inhibiting neuronal apoptosis and necrosis, thereby promoting neuronal survival. However, intracerebroventricular administration of GSK2606414 reduced p-eIF2α/eIF2α and ATF4 levels but worsened neurological outcomes by increasing pro-apoptotic proteins CHOP and caspase-12, exacerbating apoptosis and necrosis. Conversely, salubrinal suppressed CHOP and caspase-12 expression, ultimately improving neuronal survival [113].

Zimmermann *et al*. demonstrated that rapamycin-induced L-LTP failure could be reversed by the genetic removal of eIF2α kinase PERK in mice [114]. Regardless of the fact that GSK2606414 effectively reduced eIF2α phosphorylation in healthy mice, the compound did not decrease p-eIF2α in the diseased mice and, therefore, did not rescue the rapamycin-induced L-LTP failure [114].

##### 4.1.2.8 Amyotrophic Lateral Sclerosis-Frontotemporal Dementia

In an *in vitro* model of ALS/FTD, characterized by stress granule formation containing ALS-associated RNA-binding proteins, GSK2606414 reduced eIF2α phosphorylation but failed to reverse stress-induced translational arrest [84]. In the SOD1G93A murine ALS model, GSK2606414 attenuated PERK pathway gene expression, including ATF4 and GADD34. These genes serve to disinhibit protein translation while simultaneously reducing CHOP activation, thereby preventing pro-apoptotic pathways [115]. It also suppressed ATF6 and its downstream targets, such as the ER chaperones HSPA5, EDEM1, and DNAJB9, *via* a novel non-canonical signaling pathway without affecting the IRE1α pathway [115]. Despite these molecular effects, GSK2606414 failed to provide therapeutic benefits in three survival efficacy studies in SOD1G93A mice. Instead, treatment was associated with significant weight loss and reduced survival, suggesting a detrimental impact on the clinical condition of these mice [115].

Liu *et al*. demonstrated that neonatal mice exposed to sevoflurane, a common inhalational anesthetic employed in pediatric anesthesia, exhibited significant activation of the ER stress signaling pathway in the developing brain [116]. This process was accompanied by an increase in phosphorylation of PERK and eIF2α, as well as an upregulation in ATF4 and CHOP expression [116]. Pretreatment with salubrinal, an eIF2α phosphatase inhibitor, enhanced eIF2α phosphorylation and inhibited ER stress-mediated caspase-3 activation. In contrast, GSK2656157 reduced eIF2α phosphorylation but exacerbated caspase-3 activation induced by sevoflurane. Interestingly, both salubrinal and GSK2656157 attenuated sevoflurane-induced BACE-1 expression, suggesting that the BACE-1 increase may be independent of PERK-eIF2α phosphorylation [116].

Neuritin, a neurotrophic factor activated by neural activity and neurotrophins, plays a role in dendritic and axonal arbor growth as well as synaptic maturation was shown to provide neuroprotection in a mouse model of ICH by suppressing PERK and Akt signaling pathways. However, GSK2656157 counteracted the beneficial effects of neuritin by competing with this factor [117].

It can be concluded that although GSK2606414 and GSK2656157 have been demonstrated to exert favorable effects in models of diverse neurological diseases, studies have also documented pancreatic toxicity and other adverse effects resulting from the administration of these compounds. Therefore, further research is required to gain insights into the therapeutic activity of GSK2606414 and GSK2656157 in brain conditions, specifically to elucidate the underlying mechanisms of action of these compounds.

It is noteworthy that, unlike ISRIB, which broadly alleviates neuroinflammation and promotes anti-inflammatory microglial phenotypes, PERK inhibitors have been shown to increase microglial activation and polarization toward the pro-inflammatory M1 phenotype. Additionally, in certain cases, PERK inhibitors elevate pro-apoptotic proteins such as CHOP and caspase-12, exacerbating apoptosis and necrosis. These observations cautiously suggest that targeting the ISR downstream of kinase activation, such as through ISRIB, may provide a more specific and therapeutically favorable approach with improved outcomes.

#### 4.1.3 Trazodone

Trazodone, a phenylpiperazine and triazolopyridine derivative (2-[3-[4-(3-Chlorophenyl)-1-piperazinyl]propyl]-1,2,4-triazolo [4,3-a]pyridin-3(2H)-one), is an FDA-approved antagonist of serotonin type 2 (5-HT2) receptors and α-adrenoreceptors, as well as a serotonin reuptake inhibitor. It has a favorable safety profile, easily crosses the blood-brain barrier, and demonstrates anxiolytic properties and antidepressant efficacy comparable to tricyclic antidepressants, selective serotonin reuptake inhibitors, and serotonin-norepinephrine reuptake inhibitors [53, 118, 119].

In addition to its antidepressant effects, trazodone has been shown to be clinically beneficial in the treatment of a range of medical and psychiatric conditions, including behavioral disturbances associated with cognitive dysfunction, substance use disorders, post-traumatic stress disorder, obsessive-compulsive disorder, anxiety disorders, certain pain conditions, feeding and eating disorders, sexual dysfunction, insomnia, and rehabilitation after acute ischemic stroke [118]. In turn, Halliday and colleagues reported that trazodone was effective in preventing behavioral deficits, neurodegeneration, and neurological signs of prion disease in prion-infected mice without any evidence of pancreatic toxicity [53].

However, in our review, this compound was included due to its observed influence on the ISR pathway. Notably, trazodone has been shown to inhibit the phosphorylation of eIF2α, a key component of the ISR. This inhibition has been associated with neuroprotective effects in models of neurodegenerative diseases, including prion disease and tauopathy-frontotemporal dementia. In these models, trazodone treatment restored memory deficits, prevented neurodegeneration, and significantly prolonged survival, suggesting its potential as a disease-modifying treatment for dementia [53].

##### 4.1.3.1 Prion Diseases

As discussed above, trazodone was demonstrated to markedly enhance survival rates in prion-infected mice [53]. The authors elucidated that trazodone exerts its beneficial effects by inhibiting NE-induced UPR activation and eIF2α phosphorylation, thereby reversing translational attenuation and reducing ATF4 and CHOP levels in mammalian cells. Interestingly, this occurred without directly lowering p-eIF2α levels *in vitro* or *in vivo*. Trazodone restored protein synthesis in prion-infected mice by preventing a reduction in ternary complex levels caused by p-eIF2α, achieved through the modulation of ATF4 5'UTR regulation rather than acting *via* the same mechanism as ISRIB [53].

In a tauopathy-frontotemporal dementia model, trazodone also demonstrated beneficial effects, including reduced hippocampal atrophy, improved memory deficits, and decreased p-tau burden [53]. These findings suggest that trazodone’s unique mechanism of action may hold potential for treating various neurological disorders, warranting further investigation [53].

The anti-prion effects of trazodone were further validated by Albert-Gasco *et al*., who demonstrated its ability to restore synaptic and mitochondrial function during prion disease. At a stage when synapse loss was established but neuronal loss was imminent (10 weeks post-inoculation in tg37+/− mice, 18 weeks in non-canonical amino acid tagging mice), levels of key synaptic proteins, both presynaptic (*e.g*., SYN2, STX1B, SYNGR1, SYP) and postsynaptic (*e.g*., SYNGAP1, DLG4, SLC6A11), were significantly reduced by the disease process but restored with trazodone treatment [120].

Trazodone also increased levels of RAC1 and CDC42, Rho GTPases essential for F-actin assembly and synaptic remodeling, suggesting restored synaptic architecture in prion-diseased mice [120]. The treatment reversed reductions in small Rab GTPases, critical for neurotransmitter loading into synaptic vesicles, and diminished the activation of neuronal apoptotic pathways [120].

The treatment of prion-affected astrocytes with trazodone restored proteins involved in SNARE signaling (*e.g*., RAB2A, NSF, SYT3, VAMP3, ERC2), which are essential for astrocytic exocytosis [120]. This evidence aligns with the hypothesis that trazodone rescues astrocyte neurotrophic support. Furthermore, trazodone facilitated the restoration of the integrin signaling (ARF5, ARHGDIA, PFN2), essential for the tripartite synapse functionality [120].

Mitochondrial activity was also restored, with upregulation of proteins involved in glycolysis (PKM, GPI, TPI1), the tricarboxylic acid (TCA) cycle (PCX, CS, DLAT, LDHA/B), and oxidative phosphorylation (OXPHOS) complexes (NDUFS1, SDHA, UQCRC2, MT-CO2, CYC1, ATP5J2) which are diminished in prion disease [120]. Trazodone also preserved mitochondrial numbers and function, fully reversing prion-induced impairments in both neurons and dendrites [120]. Trazodone was observed to restore astrocytic mitochondrial proteins, including those involved in the TCA cycle and glycolysis (PKM, TPI1, IDH3G, SUCLG1) and OXPHOS (UQCRC1, MT-CO2, ATP5B, CYC1) [120]. This restoration of key metabolic processes in astrocytes is likely to contribute to synaptic protection [120].

Collectively, trazodone treatment reversed the synaptic and mitochondrial decline in prion-diseased mice to wild-type levels, highlighting its profound impact on ISR machinery. These findings position trazodone as a promising therapeutic candidate for proteinopathies like Alzheimer’s disease, where ISR dysregulation contributes to pathology.

##### 4.1.3.2 Alzheimer's Disease

The anti-AD effects of trazodone were reported by the de Oliveira research group, which demonstrated that chronic treatment with trazodone reduced the activation of cellular components that are key to neuroinflammation and tau pathology in rTg4510 mice when tau aggregation is observed [121]. Furthermore, trazodone was demonstrated to rectify sleep disturbances and enhance olfactory-dependent memory, which bears a resemblance to the initial indications observed in patients diagnosed with AD or FTD [121]. The authors demonstrated that trazodone induced an AD-mediated reduction in microglial activation, as evidenced by a decrease in the expression of IBA1 and NLRP3 inflammasome and an accumulation of non-phosphorylated tau protein [121]. The authors, therefore, propose that trazodone may improve tau pathology *via* the inhibition of the microglial NLRP3 inflammasome [121]. It was demonstrated that trazodone reduced the UPR effector ATF4 in the cortex of rTg4510 mice without affecting the upstream UPR-dependent event, as shown in the hippocampus [121]. Moreover, the authors observed a robust correlation between the expression of the NLRP3 inflammasome effector caspase-1 and phosphorylated p38 and ATF4, as well as between phosphorylated p38 and ATF4 [121]. The authors, therefore, propose that the effects of trazodone are mediated by the inhibition of p38 MAPK phosphorylation through the antagonism of G-protein-coupled receptors expressed on microglia [121]. This subsequently resulted in a reduction in ATF4 levels, which occur downstream of the PERK/eIF2α UPR-dependent pathway [121]. A decrease in phosphorylated p38 and ATF4 expression leads to the downregulation of NLRP3 inflammasome components, including ASC, pro-caspase-1, and caspase-1 [121].

Trazodone was found to enhance olfactory memory during the first month of treatment but had no effect on spatial working memory in rTg4510 mice [121]. In conclusion, the results of this study suggest that trazodone may represent a promising avenue for therapeutic intervention in the progression of tauopathy and its associated early symptoms.

In summary, the extant studies describe highly favorable effects of trazodone in the treatment of various neurological disorders. Indeed, this compound possesses numerous advantages and is both safe and efficacious [118, 119]. Nevertheless, numerous studies have documented the beneficial effects of trazodone in neurological models without evaluation of alterations in the ISR signaling pathway [122-124]. For instance, Daniele *et al*. demonstrated that trazodone is capable of alleviating LPS/TNFα-induced inflammation in neuronal-like cells through the suppression of pro-inflammatory factors expression (IL-6, IFN-γ, NF-κB, p38, JNK) and the enhancement of anti-inflammatory factors expression (IL-10, BDNF, CREB) [122]. However, the authors did not investigate whether these effects occurred as a consequence of trazodone's ability to modulate ISR signaling [122]. The absence of ISR signaling characterization in these studies may be attributed to the fact that the research group led by Halliday has only recently described the capacity of trazadone to inhibit ISR [53]. Conversely, evaluating the therapeutic efficacy of trazodone without accounting for its antidepressant and other therapeutic effects is not feasible. Its ability to modulate multiple signaling pathways could represent a valuable combination for developing a versatile therapeutic agent. Nevertheless, in order to establish a robust basis for clinical trials, it is essential to conduct a more comprehensive and detailed characterization of the effects of trazodone, encompassing the assessment of alterations in distinct signaling pathways it may influence.

The effects of ISR inhibitors on various neurological diseases are summarized in Fig. (**2**).

### 4.2 ISR Activators in Neurological Diseases

As it was discussed above, ISR inhibitors can effectively mitigate synaptic dysfunction, exacerbation of cognitive decline and neuronal loss by restoring protein homeostasis and neuronal function. However, an interesting aspect of the ISR is that both its activators and inhibitors offer therapeutic potential for the treatment of neurological diseases. While chronic activation of the ISR can lead to detrimental effects, transient activation of ER-stress and UPR signaling pathways has been demonstrated to exert a beneficial influence on the course of numerous neurological diseases [90, 94, 97, 100, 104]. It can be hypothesized that the effect of an ISR modulator is dependent on the stage of the neurological disease in which it is applied. This hypothesis may provide an explanation for the existence of reports presenting contradictory results following the application of ISR inhibitors to the same disease models. Moreover, this hypothesis may explain the numerous studies that have reported the beneficial effects of ISR activators in treating neurological diseases. This hypothesis is corroborated by the evidence, that PERK kinase is frequently attributed with a range of protective functions [125]. ISR-related transcription factor ATF4 has also been demonstrated to safeguard differentiated PC12 cells against neuronal death in PD models [126]. Consequently, we posit that an account of the role of ISR activators in their potential use as therapeutic agents in neurological diseases is essential. The following chapter will delve into the therapeutic effects of ISR activators in various neurological disorders, highlighting their potential roles and mechanisms.

#### 4.2.1 Salubrinal

Salubrinal (3-phenyl-N-[2,2,2-trichloro-1-[[(8-quinolinyl- amino)thioxomethyl]amino]ethyl]-2-propenamid) is a selective inhibitor of GADD34/PP1 and CReP/PP1 phosphatases complexes that dephosphorylate p-eIF2α [54]. Salubrinal crosses the blood-brain and ophthalmic barriers [127-129]. Given the pivotal role of ER stress in the pathogenesis of neurological diseases, salubrinal may represent a promising therapeutic option for these conditions [130]. Furthermore, numerous studies have defined salubrinal as an “endoplasmic reticulum stress inhibitor” [131, 132]. It is, therefore, essential to describe the effects of salubrinal in the treatment of various neurological disorders, with a particular focus on the findings of existing studies.

##### 4.2.1.1 Alzheimer’s Disease

The pathological hallmarks of AD include the accumulation of the amyloid-β peptide in the brain and of neurofibrillary tangles comprising hyperphosphorylated tau protein, along with neuroinflammation, synapse failure/loss, and neurodegeneration [66]. These ultimately result in memory failure [66]. A study by Huang and colleagues demonstrated that salubrinal reduced the Aβ1-42 peptide-induced death of rat primary cortical neurons by downregulating the NF-κB pathway without affecting ER stress [133]. Surprisingly, in this study, salubrinal did not influence eIF2α phosphorylation during the six-hour treatment period [133]. Salubrinal has been shown to mitigate AD-associated microglial activation and cell death, as evidenced by a reduction in IL-1B and cleaved caspase 3 levels [133]. Salubrinal's inhibitory effects on IKK activation, IκB degradation, and NF-κB activation were demonstrated to be the underlying mechanisms driving this outcome [133]. It was demonstrated that the Grp78/Bip and eIF2α PERK-associated pathways exert a protective effect in Aβ-mediated neuronal cell death since the addition of salubrinal to Aβ1-42-treated cells stimulated these pathways, enhancing cell viability by reducing caspase-4 and caspase-3 activity [134]. Shim *et al*. demonstrated that FOXRED2, a FAD-dependent oxidoreductase domain-containing protein that is localized in the ER, is associated with the unfolded protein response and is upregulated under exposure of rat cortical neurons and SH-SY5Y cells to Aβ42, leading to the inhibition of proteasome activity and the accumulation of ubiquitin-conjugated proteins, a hallmark of AD [135]. The ectopic expression of FOXRED2 has been demonstrated to induce ER stress-mediated cell death *via* caspase-12, a process that can be reversed by salubrinal [135]. In an AD-like rat brain model, Goswami and colleagues demonstrated that Aβ1-42 treatment resulted in a multifaceted response comprising increased cell death, accompanied by elevated caspase-3 and caspase-12 activity, ER stress activation, and ISR pathway suppression, as indicated by reduced levels of p-eIF2α [136]. The Aβ1-42-induced effects were partially but significantly reversed by salubrinal treatment [136]. The authors suggest that ER stress is strongly implicated in AD pathogenesis and could be alleviated with salubrinal treatment [136]. In summary, these findings indicate that salubrinal treatment exerts beneficial effects on AD pathology, as evidenced by the alleviation of ER stress, enhancement of cell viability, and reduction of inflammatory responses.

##### 4.2.1.2 Traumatic Brain Injury

TBI represents the most prevalent neurological disorder, encompassing a wide range of short- and long-term physical, cognitive, and emotional impairments that are partially contingent upon the severity of the injury [137]. The neuroprotective effects of salubrinal in a murine model of mild traumatic brain injury (mTBI) have been demonstrated by Rubovitch *et al*. to be achieved by maintaining the levels of p-eIF2a, but not the total ATF4, after mTBI, that is evidenced by the significant reduction in p-eIF2α protein levels observed in the post-mTBI ipsilateral cortex [137]. In our opinion, this reduction is open to question, given that the authors did not undertake a comparison of p-eIF2α/eIF2α ratios but rather compared the p-eIF2α levels normalized to tubulin [137]. Treatment with salubrinal was demonstrated to alleviate the cognitive impairments observed in both visual and spatial memory following mTBI, in addition to reducing the number of degenerating neurons in the cortices of the injured mice [137]. On the other hand, ER stress and activation of the ISR signaling pathway occurred in the mice cortex and hippocampus following TBI [138]. This was evidenced by an increase in the levels of proteins GRP78, CHOP, and p-eIF2α [138]. Furthermore, CHOP was co-expressed with neurons, astrocytes, microglia, vascular endothelial cells, and pericytes in the post-TBI brain, collectively forming the neurovascular unit [138]. Post-TBI treatment with salubrinal preserved cellular membrane integrity facilitated motor recovery, enhanced learning and memory, and led to a reduction in brain lesion volume [138]. The protective mechanisms of salubrinal on TBI, as proposed by the authors, may be associated with the suppression of ER stress, as evidenced by the downregulation of ATF4, GRP78, and CHOP, and reduction in apoptotic cell death through a decline in caspase-12 and caspase-3 cleavage, as well as in Bcl-2 [138]. The findings of the Tan research group are consistent with those of the previous study, confirming that TBI activates ER stress and ISR signaling, leading to the upregulation of p-PERK, p-eIF2α, ATF4, spliced XBP-1, GRP78, and cleaved ATF6 [139]. Additionally, the research group has demonstrated that TBI enhances apoptotic cell death by upregulating GADD153, caspase-3, and caspase-12 [139]. Salubrinal treatment was observed to diminish brain damage and mitigate both ER and mitochondrial damage in neurons derived from the injured cortex while also reducing ROS production, ER stress activation, and apoptosis in post-TBI models [139]. The authors posit that salubrinal exerts its beneficial effects in their study through the alleviation of ER stress, for instance, by stimulating the ISR pathway [139]. Logsdon *et al*. reported concordant findings, demonstrating that treatment of TBI rats with salubrinal alleviated ER stress (by reducing JNK phosphorylation and CHOP activation), oxidative stress (observed by reduced levels of carbonyl and superoxide), neuroinflammation (by reducing TNFα, IL-1β, iNOS and NF-κB p65 expression) and neurodegeneration, as well as ameliorating impulsive-like behavior [140].

The Zhang research group also demonstrated comparable effects in an ICH model [113]. Following the induction of ICH, the activation of the ISR pathway was observed, accompanied by a notable increase in cell death [113]. The neurological deficits and edema induced by ICH were found to be ameliorated when the PERK pathway was activated using salubrinal, and conversely, these effects were aggravated by the blockade of the PERK pathway using GSK2606414 [113]. In light of these findings, the authors propose that PERK exerts a neuroprotective effect in their model [113]. Collectively, treatment with salubrinal was demonstrated to mitigate TBI pathology through the alleviation of oxidative and ER stress, the reduction of inflammatory responses, and the enhancement of cell viability.

##### 4.2.1.3 Parkinson's Disease

Parkinson's disease is a progressive neurodegenerative disorder that is characterized by the death of dopaminergic neurons in the substantia nigra and the abnormal accumulation of α-synuclein (α-Syn) [141, 142]. Such filamentous accumulations in neuronal perikarya and processes are designated as Lewy bodies and Lewy neurites, respectively [141]. Colla *et al*. demonstrated that treating the A53TαS Tg mouse model and the rat AAV2/6 model of α-synucleinopathy with salubrinal markedly delays the onset of motoric symptoms and reduces the accumulation of αS oligomers *in vivo* [143]. Nevertheless, salubrinal treatment did not enhance the survival of striatal dopaminergic neurons nor influence the progression of the disease in the animals [143]. In the rat model of rotenone-induced Parkinsonism, Gupta *et al*. observed elevated levels of ER-stress (GADD153 and GRP78) and proapoptotic (caspase 3 and caspase 12) proteins [144]. The administration of salubrinal to rats treated with rotenone resulted in the reversal of these changes, accompanied by a reduction in DNA fragmentation and neuronal degeneration [144]. However, salubrinal did not affect the elevation (ATF4, p-PERK, XBP1, p-IRE1α, ATF6) or decrease (p-eIF2α) levels of some proteins [144]. Collectively, the authors posit that alterations in eIF2α play a pivotal role in their disease model, as evidenced by the alleviation of PD-like symptoms through the reduction of ER stress and neuronal death following salubrinal treatment [144]. The findings were corroborated by the Wu research group, demonstrating that salubrinal alleviates rotenone-induced death of SH-SY5Y neuroblastoma cells through the downregulation of CHOP and C/EBPβ isoform LIP [142]. However, the authors reveal that the protective effect of salubrinal in their model is dependent on the enhancement of ATF4 and parkin protein expression [142].

In the LPS-induced hemi-Parkinson rat model, Cankara and colleagues demonstrated that treatment with salubrinal attenuates the disease's symptoms, including forelimb akinesia, dopaminergic desensitization, bradykinesia and motor impairment [145]. Furthermore, salubrinal was demonstrated to alleviate LPS-induced neuroinflammation, as evidenced by a reduction in TNF-α, IL-6, and IL-1β protein levels [145]. Additionally, it was shown to suppress caspase-3, caspase-9, DUSP, and PP1 protein expression, thereby attenuating LPS-induced neuronal death [145].

In conclusion, the majority of studies utilizing models of neurological diseases have reported favorable effects of salubrinal treatment. Some studies have revealed an unexpected outcome whereby treatment with salubrinal, an ISR activator, has been observed to result in a reduction in ATF4 and the pro-apoptotic factor CHOP, major effector molecules of the ISR [138, 140, 146, 147]. Only a few studies have demonstrated that salubrinal treatment stimulates cell death by upregulating the expression of CHOP and cleaved caspase-12 [95, 148]. It was also shown, that treatment of healthy C57BL/6 mice with salubrinal impaired long-term memory in both novel object recognition and contextual fear conditioning tests [66]. These changes can be reversed by the ISRIB administration [66]. Nevertheless, this is outweighed by the substantial body of research indicating the neuroprotective properties of salubrinal. For example, treatment with salubrinal has been demonstrated to exert neuroprotective effects in the context of status epilepticus, global cerebral ischemia, spinal cord injury, chronic stress-induced glutamatergic neuronal damage, hereditary spastic paraplegia, TDP-43 neuronal toxicity, ischemic stroke, and cortical stab injury [128, 129, 146, 147, 149-154]. Furthermore, salubrinal has been shown to have minimal or no cardiotoxic or hepatotoxic effects [155]. We posit that these findings pave the way for salubrinal to enter clinical trials as a promising drug. It is also essential to direct attention to the few reports of detrimental effects associated with salubrinal treatment in order to ascertain the underlying cause and enable a comprehensive understanding of the mechanism of action and potential off-target effects of the compound.

#### 4.2.2 Guanabenz

Guanabenz (2,6-dichlorobenzylidene aminoguanidine) is an FDA-approved centrally acting selective α-2 adrenergic agonist and antihypertensive drug that has also been shown to readily cross the blood-brain barrier and downregulate inflammatory responses *via* both the regulation of the ISR pathway and p-eIF2α-independent signaling [156, 157]. Indeed, over a decade ago, Tsaytler and colleagues demonstrated that guanabenz exerts an ISR stimulatory effect through the suppression of the stress-inducible regulatory subunit of PP1, GADD34, thereby preventing p-eIF2α dephosphorylation [55]. Later, Carrara *et al*. corroborated the selectivity of guanabenz towards GADD34 [158]. The authors demonstrated that guanabenz is an allosteric inhibitor of GADD34 that binds selectively to its amino-terminal region, thereby inducing a conformational change [158]. Consequently, the function of the regulatory subunit is compromised, and dephosphorylation of the substrate is inhibited [158]. Given the specificity of the compound and the involvement of ISR signaling in various brain conditions, it is important to describe the potential therapeutic role of guanabenz in neurological diseases.

##### 4.2.2.1 Alzheimer's Disease

In rodent models of AD, Singh and colleagues found that guanabenz treatment provided substantial protection against AD-specific behavioral and pathological markers, including alterations in acetylcholinesterase activity, tau phosphorylation, amyloid precursor protein processing, and memory retention [159]. Furthermore, guanabenz has been demonstrated to alleviate oxidative stress and other alterations associated with AD, including impaired mitochondrial function (decreased mitochondrial membrane potential, cytochrome-c release, ATP reduction, and decreased complex I activity), endoplasmic reticulum stress (elevated GRP78 and GADD153, increased cleaved caspase-12), neuronal cell death (decreased Bcl-2, increased Bax, elevated cleaved caspase-3), and DNA fragmentation [159]. The authors propose that guanabenz exerts a broad protective influence on a multitude of disease-linked degenerative markers and signaling pathways [159]. However, the authors of the study did not demonstrate whether these effects are mediated by modulation of the ISR pathway [159].

##### 4.2.2.2 Experimental Autoimmune Encephalomyelitis - Multiple Sclerosis

In a rodent experimental autoimmune encephalomyelitis (EAE) model of multiple sclerosis (MS), Way *et al*. showed that guanabenz effectively protected oligodendrocytes from inflammatory cytokine-induced death *in vitro*, *ex vivo*, and *in vivo* [160]. Furthermore, guanabenz treatment in chronic and relapsing-remitting EAE mice resulted in a delay in disease onset and a reduction in relapse severity, respectively [160]. Moreover, the authors discovered that guanabenz exerts a protective effect on oligodendrocytes that is distinct from its potential role in modulating the immune response [160]. The administration of the compound was observed to confer a protective effect on oligodendrocytes against damage induced by interferon-γ despite the absence of adaptive immune responses in the experimental models [160]. The authors observed that the guanabenz-mediated enhancement of the ISR is sufficient to protect oligodendrocytes from inflammation-induced loss [160]. This results in a reduction in apoptotic cells and myelin debris, which are essential for maintaining an inflammatory response [160]. This may explain the reduced number of CD4+ T cells observed in the central nervous system (CNS) of guanabenz-treated mice [160]. In a mouse model of EAE, guanabenz markedly diminished the inflammatory responses of microglia and macrophages, resulting in a reduction in the number of T cells, the primary disease-causing cells in this model [161]. Nevertheless, when guanabenz was administered to a cuprizone-induced demyelination model where immune cells play a less prominent role, the authors observed no discernible improvements in remyelination or augmentations in oligodendrocyte counts, and its impact on microglial activity was comparatively limited [161]. In light of these findings, it appears that guanabenz may serve as a promising therapeutic candidate for the mitigation of neuronal inflammation, with a relatively low risk of adverse effects [161].

##### 4.2.2.3 Amyotrophic Lateral Sclerosis

In a rodent model of ALS, Wang and colleagues demonstrated that guanabenz treatment resulted in a notable delay in disease onset, an extension of the early disease phase, and an increase in survival rates in G93A mice [162]. Furthermore, the neuropathological changes observed in guanabenz-treated G93A mice occurred at a later stage than in the untreated control group [162]. The administration of guanabenz to G93A mice was observed to result in a reduction in the quantity of mutant SOD1 (mtSOD1) present in the lumbar spinal cord when compared to untreated G93A mice of a similar age [162]. The authors hypothesize that the reduction in total mtSOD1 in guanabenz-treated G93A mice is a consequence of the enhanced phosphorylation of eIF2α resulting from the inhibition of GADD34 [162]. The enhanced phosphorylation of eIF2α presumably resulted in a reduction in total mtSOD1, either due to a decrease in the synthesis of mtSOD1 or an increase in the clearance of mtSOD1 aggregates, potentially as a consequence of increased expression of cytoprotective genes [162]. Nevertheless, the authors did not rule out the possibility that guanabenz exerts its beneficial effects on the disease process through the modulation of pathways other than the ISR/UPR [162]. This is based on the observation that the levels of p-eIF2α remained unaltered in guanabenz-treated G93A mice when compared to untreated G93A mice during the early stages of the disease [162]. Only the end-stage guanabenz-treatment group demonstrated an increase in p-eIF2α, whereas no such elevation was observed in the end-stage untreated group [162]. The authors hypothesized that enhanced phosphorylation of eIF2α did, in fact, occur as a result of both guanabenz treatment and GADD34 mutation [162]. However, they suggest that early in the disease, this is a rapid and transient response, triggering a relative decrease in ER stress and unfolded proteins [162]. This results in a lower level of PERK activation and eIF2α phosphorylation [162]. The observed increase in p-eIF2α in guanabenz-treated G93A mice is proposed to be the underlying mechanism responsible for the reduction in total mtSOD1 levels compared to similarly aged untreated G93A mice [162]. This is achieved through the enhanced suppression of protein translation, including mtSOD1, and the reduction in mtSOD1 aggregation [162]. In instances where the ER stress response is overwhelmed by the UPR, eIF2α phosphorylation is increased, as observed in end-stage guanabenz-treated G93A mice [162].

In a parallel study utilizing the identical rodent model of ALS, Jiang *et al*. corroborated these observations and demonstrated that guanabenz markedly postponed the advent of disease symptoms, extended survival, augmented motor performance, decelerated body weight loss, and prolonged motor neuron survival in the lumbar spinal cord of female SOD1 G93A mice [163]. The administration of guanabenz resulted in a significant increase in the p-eIF2α without any alteration in the overall protein abundance of eIF2α [163]. Furthermore, guanabenz was observed to downregulate the levels of the ER chaperone BiP, ATF6, and IRE1, which are markers of two different ER stress pathways [163]. The administration of guanabenz led to the concomitant upregulation of the anti-apoptotic protein Bcl-2 and downregulation of pro-apoptotic proteins CHOP, BAX, and cytochrome C in SOD1 G93A mice, thereby suggesting a protective effect against apoptosis [163]. The observed elevation in phosphorylated eIF2α induced by guanabenz treatment is hypothesized to mitigate motor neuron death by reducing endoplasmic reticulum and mitochondrial stress [163]. The downregulation of BiP indicates that guanabenz has the capacity to diminish ER stress by reducing protein misfolding and mitigating neuronal damage through CHOP downregulation, thereby preserving a greater number of neurons [163]. The authors attributed the observed improvement in motor performance to the presence of a greater number of preserved motor neurons and the delay in body weight loss to the decreased loss of neurons and increased ability to move in the experimental animals [163].

The favorable outcomes observed in rodent models of ALS following the administration of guanabenz have paved the way for the compound to progress to phase 2 clinical trials conducted by Dalla Bella and colleagues as a potential anti-ALS drug [164]. The primary assessment of efficacy was conducted using an intention-to-treat analysis [164]. The administration of either 64 mg or 32 mg of guanabenz, either as a standalone treatment or in combination, resulted in a notable reduction in the proportion of patients who progressed to more advanced stages of ALS at the six-month mark, when compared to the anticipated rate under the non-futility hypothesis [164]. Furthermore, the median rate of decline in the total revised ALS Functional Rating Scale score was significantly slower in patients who received treatment [164]. The protective effect of guanabenz was particularly pronounced in patients with bulbar onset ALS, as none of the treated patients in this group progressed to a more severe stage of the disease at six months, in contrast to 50% of those receiving placebo [164]. The incidence of any adverse events was found to be statistically significantly higher in all guanabenz-treated groups in comparison to the placebo group [164]. The administration of higher doses of guanabenz was associated with a significantly greater prevalence of drug-related adverse effects [164]. Furthermore, the group receiving 64 mg of the drug exhibited a significantly elevated rate of withdrawal from the study due to these adverse effects [164]. The incidence of serious adverse events was comparable between the guanabenz-treated patients and those who received a placebo [164]. The alpha-2 adrenergic properties of guanabenz were evident in this study, resulting in a markedly elevated dropout rate in the 64 mg and 32 mg dosing arms relative to the placebo and other treatment groups [164]. The ability of guanabenz to induce hypotension in non-hypertensive patients clearly limits its practical application in further assessment of ALS [164].

##### 4.2.2.4 Epilepsy Model

Biggane and colleagues evaluated several compounds for the reduction of hippocampal CA3 epileptiform activity in rats [165]. The authors found that dexmedetomidine, an imidazoline (potency (pEC50), 8.59; relative efficacy (RE), 67.1%), and guanabenz, a guanidine (pEC50, 7.94; RE, 37.9%), had the highest potency (pEC50) compared to norepinephrine (pEC50, 6.20; RE, 100%) [165]. In contrast, the catecholamines epinephrine (pEC50, 6.95; RE, 120%) and a-methyl-norepinephrine (pEC50, 6.38; RE, 116%) were the most potent, but none can cross the blood-brain barrier due to a highly charged protonated amine [165].

##### 4.2.2.5 Traumatic Brain Injury

In a study conducted by Dash and colleagues, TBI was observed to elevate the levels of p-eIF2α in both the CA1 and CA3 regions of the hippocampus in rats [166]. Administration of guanabenz reduced the severity of TBI-induced deficits in motor function, vestibular-motor coordination, recognition memory, and spatial learning [166]. Moreover, the guanabenz group showed a reduced volume of cortical lesions and a decrease in neuronal damage in the hippocampus, both of which are consequences of TBI [166]. The authors, therefore, propose that an increase in p-eIF2α may prove beneficial in the context of TBI [166]. The autosomal-dominant disorder observed in tuberous sclerosis complex (TSC) is caused by TSC1/2 loss-of-function mutations, which manifest in neurodevelopmental deficits, including profound hypomyelination and oligodendrocyte loss [167]. The findings of Jiang *et al*. indicate that the loss of TSC1 in murine oligodendrocyte lineage cells results in widespread oligodendrocyte death in the CNS by activating the prominent PERK/eIF2α-mediated ER stress and Fas/JNK apoptotic pathways [167]. The sustained endoplasmic ER stress in TSC1 mutants triggers apoptotic programs upon oligodendrocyte differentiation [167]. The treatment of guanabenz in a model of TSC1 mutants, which exhibits widespread oligodendrocyte loss, has been demonstrated to elevate the levels of p-eIF2α and induce protective adaptive responses [167]. This elevation effectively protected oligodendrocytes from apoptosis and maintained a balance between stress mitigation and cell death [167].

##### 4.2.2.6 Parkinson's Disease

Neuronal exposure to 6-OHDA, a neurotoxin commonly used to model Parkinson's disease-related features, markedly enhanced GADD34 expression [168]. The localization of GADD34 is significantly altered in dopaminergic neurons of the substantia nigra in PD cases [168]. The administration of guanabenz was observed to effectively safeguard dopaminergic neurons from death induced by 6-OHDA in differentiated PC12 cells, primary neurons isolated from the ventral midbrain, and the substantia nigra of mice [168]. *In vitro* studies have demonstrated that guanabenz treatment enhances the phosphorylation status of eIF2α and elevates the levels of ATF4 and parkin proteins in response to 6-OHDA exposure [168]. Furthermore, the protective effects of guanabenz against 6-OHDA-induced neuronal death were abolished when either ATF4 or parkin was silenced *in vitro*, indicating their involvement in the drug's protective mechanisms [168]. Similar protective effects were observed when guanabenz was administered to primary cultures of cortical neurons treated with camptothecin, a drug that induces neuronal death through a mechanism involving topoisomerase I inhibition [168]. This protective effect was dependent on both ATF4 and parkin proteins [168]. It has been demonstrated that prolonged exposure of neurons to mitochondrial stress results in the accumulation of reactive oxygen species (ROS) and the disruption of proteostasis, which in turn leads to the activation of Tau protein aggregation [169]. In conditions of long-term mitochondrial stress, the inhibition of the ISR signaling pathway using ISRIB resulted in the accumulation of Tau dimers [169]. The administration of the ISR pathway activators salubrinal, guanabenz, or sephin1 in this context resulted in a partial reduction in the formation of Tau aggregates, indicating the potential of these agents to prevent the initial stages of Tau aggregation [169]. However, the effect of ISR activation on the reduction of Tau dimerization was subtle, and as the authors highlighted, its physiological significance must be clarified in future studies [169].

##### 4.2.2.7 Leukoencephalopathy

Vanishing white matter (VWM) is a leukodystrophy characterized by chronic neurological deterioration and episodes of rapid decline provoked by stresses, especially febrile infections [170]. Neuropathological examination reveals white matter rarefaction and cystic degeneration, accompanied by feeble astrogliosis, deficient myelin, immature astrocytes, and oligodendrocytes [170]. There is currently no cure for VWM, and patients inevitably succumb to the disease prematurely [170]. The underlying mechanism of VWM is attributed to recessive mutations in the genes that code for the subunits of eIF2B, which ultimately results in a reduction in the function of this critical protein complex, which is essential for protein translation [170]. Given the involvement of p-eIF2α signaling perturbations in VWM pathogenesis, ISR modulators are widely tested as potential therapeutic agents for VWM, with ISRIB representing a particularly promising candidate [170-172]. 2b5^ho^ mice and VWM patient brains display constitutive enhanced expression of ATF4-regulated mRNAs, which ISRIB is able to suppress by partly normalizing eIF2B activity [170]. This results in improvements to white matter and astrocyte pathology, as well as an amelioration of the clinical disease in VWM models [170]. Intriguingly, the research groups of Witkamp and Dooves have demonstrated that guanabenz, a compound with effects that are opposite to those of ISRIB, has the capacity to alleviate VWM pathology in many aspects [171, 172]. Furthermore, Witkamp *et al*. demonstrated that guanabenz treatment results in a reduction in p-eIF2α levels and the inhibition of the ATF4-driven gene expression program in the cerebellum neurons of VWM mice [171]. However, the authors did not elucidate the mechanism by which guanabenz treatment led to the decrease in excessive eIF2α phosphorylation in VWM mice [171].

##### 4.2.2.8 Spinal Cord Injury

Nevertheless, the effect of guanabenz in the treatment of neurological diseases remains incompletely understood, and the available evidence does not permit a definitive conclusion as to its safety and efficacy. For instance, in the murine model of SCI, Saraswat Ohri *et al*. demonstrated that treatment with guanabenz *in vivo* did not result in any reduction of locomotor deficits post-SCI [173]. It was observed that the treatment attenuated GADD34 and XBP1 transcript levels but had no significant effect on ATF4 and CHOP transcript levels in mouse oligodendrocyte precursor cells (mOPCs) post-SCI [173]. The levels of p-eIF2α remained elevated at 24 hours post-treatment with guanabenz, which the authors hypothesize may have a detrimental impact on the survival of mOPCs [173]. Taken together, the authors observed that guanabenz was unable to facilitate functional recovery following SCI [173]. In contrast to the findings of the previous study, Bisicchia and colleagues reported that post-SCI treatment with guanabenz protected rat neurons from the detrimental effects of unresolved endoplasmic reticulum stress by promoting the restoration of autophagic flux through increased lysosomal biogenesis [174]. This was associated with enhanced activation of transcription factor EB, a key regulator of cellular autophagy and lysosome biogenesis, which resulted in improved neuronal survival and functional recovery following SCI [174]. Furthermore, these effects were observed to persist even after the cessation of the treatment [174]. The authors reported that SCI-induced ER stress alters transcription factor EB activity in remote neurons, which was restored by guanabenz through unknown mechanisms [174].

The application of guanabenz in a mouse model of ALS resulted in disease progression despite promoting the viability of fibroblasts expressing the disease-causing G93A mutant SOD1 protein under tunicamycin-induced ER stress conditions [175]. Vieira and colleagues evaluated alterations in the levels of Atf4, Bcl2, Bip, Ccnd1, and Chop transcripts in guanabenz-treated mice and observed that guanabenz significantly decreased only the Chop level [175]. The authors posit that the deleterious effect of guanabenz observed in their study may be attributed to the use of male mice as a model, given that previous work by Jiang *et al*. utilized female mice as a model for ALS and reported beneficial anti-ALS effects of guanabenz [163, 175]. Besides that, the toxicity of guanabenz remains a topic of contention. For instance, Abdulkarim *et al*. demonstrated that guanabenz induced β-cell dysfunction *in vitro* and *in vivo* in rodents, resulting in impaired glucose tolerance [176]. The cytotoxic effects of free fatty acids on β-cells of pancreatic islets from rats and humans were significantly enhanced by guanabenz [176]. The findings revealed that guanabenz augmented the effects of free fatty acids on the phosphorylation of eIF2α and the expression of the pro-apoptotic gene CHOP, which was identified as the key mediator of the enhanced sensitivity to lipotoxicity [176]. The study findings demonstrate that guanabenz does not confer protection against ER stress in β-cells [176]. Rather, it has been observed to enhance the detrimental effects of lipotoxicity through the activation of the PERK/eIF2α/CHOP signaling pathway [176]. Taken together, further investigation is required into the mechanism of action of guanabenz in various neurological diseases. However, in any case, the antihypertensive activity represents a significant obstacle to the specific application of this compound in the modulation of ISR in various brain conditions.

#### 4.2.3 Sephin1

Selective inhibitor of a holophosphatase (Sephin1; 2-(2-chlorobenzylidene)hydrazinecarboximidamide) is a guanabenz derivative that is able to cross the blood-brain barrier and selectively disrupts the GADD34-PP1 complex while sparing the related CREP-PP1 complex [56]. Furthermore, it has been demonstrated that this agent does not elicit any discernible α2-adrenergic side effects in either in *vitro* or *in vivo* models [56]. Sephin1 was developed by the Das research group, which demonstrated that chronic administration of Sephin1 showed protective effects in Charcot-Marie-Tooth disease and in the SOD1G93A mouse model of ALS [56]. These effects included the rescue of myelination, a decrease in the expression of ER-stress genes, and the prevention of motor defects [56]. Moreover, in a rodent model of AD, Maltsev, and colleagues demonstrated that sephin1 is capable of PP1α blockade during Aβ25-35-treated hippocampal slice incubation, thereby preventing Aβ25-35-mediated L-LTP suppression [177]. Furthermore, sephin1 was observed to restore paired-pulse facilitation ratios to control levels at interstimulus intervals of 30-100 ms [177]. Thus, the work of Das and colleagues has paved the way for further investigation of Sephin1 as a potential drug for various neurological diseases.

##### 4.2.3.1 Charcot-Marie-Tooth Disease

Sephin1 is a promising therapeutic agent for alleviating peripheral neuropathy in a mouse model of Charcot-Marie-Tooth disease type 1B (CMT1B), as evidenced by both *in vitro* and *in vivo* studies [178]. *In vitro*, sephin1 was observed to enhance myelination in CMT1B dorsal root ganglia explant co-cultures [178]. The long-term administration of sephin1 in CMT1B mice resulted in notable functional improvements, as evidenced by enhanced motor performance, restored nerve conduction velocity, and structural preservation of peripheral nerves [178]. Furthermore, treatment with sephin1 was observed to significantly reduce ER stress and to lower the expression of c-Jun, a negative regulator of myelination, in the cells [178]. The administration of sephin1 resulted in an improvement in motor capacity, neurophysiology, and peripheral nerve morphology, as well as a partial adjustment in both myelin protein stoichiometry and stress levels in a murine model of Charcot-Marie-Tooth disease type 1A [178].

Taken together, the authors posit that sephin1 has beneficial effects in Charcot-Marie-Tooth disease and hypothesize that by prolonging the phosphorylation of eIF2α, sephin1 attenuates the translation of highly expressed myelin proteins, including peripheral myelin protein 22, thereby restoring stress levels to those observed in wild-type mice [178].

##### 4.2.3.2 Multiple Sclerosis

Pernin and colleagues demonstrated that activation of ISR signaling occurs in oligodendrocytes in response to MS-like conditions [179]. Treatment with sephin1 resulted in enhanced cell death under low glucose MS-like culture conditions and increased expression of ATF4, CHOP, and p-eIF2α in the cells [179]. Conversely, treatment with ISRIB led to a reduction in ATF4 and CHOP expression but did not affect the phosphorylation of eIF2α in the cells [179]. Furthermore, ISRIB was observed to restore process outgrowth under conditions of cellular stress [179]. The administration of the compound to cells that had already undergone stress was found to reduce delayed cell death and extend the period during which recovery could occur [179]. The authors posit that evaluation of the status of the ISR pathway may prove beneficial in the selection of an anti-MS drug [179]. Further elucidation of the ISR role in MS pathogenesis brought the Chen research group, which has conducted a series of studies aimed at investigating the effects of sephin1 in MS pathology [180-182]. The authors demonstrated that treatment with Sephin1 significantly delayed the onset of EAE in mice, and when combined with interferon-β, it showed an additional protective effect against disease progression [180]. Subsequent experiments demonstrated that Sephin1 confers protection to oligodendrocytes, axons, and myelin in EAE mice, which is associated with prolonged activation of the ISR pathway and reduced CNS inflammation [180]. Furthermore, the authors showed that the protective effect of Sephin1 is not attributable to alterations in T cell priming in the peripheral immune system and that the therapeutic target of GADD34 inhibition resides within the CNS [180].

In a rodent model of multiple sclerosis, treatment with both Sephin1 and the oligodendrocyte differentiation enhancer bazedoxifene (BZA) resulted in a significantly accelerated process of remyelination, compared to either treatment alone, in the presence of the inflammatory cytokine interferon-γ [181]. As the authors propose, the observed synergistic effect of Sephin1 and BZA on remyelination can be attributed to their complementary mechanisms of action on oligodendrocyte progenitor and mature oligodendrocyte populations [181]. It was demonstrated that BZA stimulates the differentiation of oligodendrocyte precursor cells in the presence of interferon-γ, while Sephin1 enables the survival and development of oligodendrocytes under the same conditions [181]. The administration of Sephin1 was demonstrated to prolong the duration of interferon-γ-induced ISR by increasing the levels of p-eIF2α, resulting in a reduction in global protein synthesis and the accumulation of RNA stress granules [181]. Additionally, the authors demonstrated that an ISR suppressor, 2BAct, which reverses the effects of p-eIF2α, can partially inhibit the protective effects of Sephin1 on EAE [181]. This indicates that Sephin1 protects oligodendrocytes against inflammation by enhancing the ISR [181]. Taken together, the authors propose that the combined administration of Sephin1 and BZA facilitates more expeditious and robust remyelination in the context of inflammation [181].

This is of paramount importance, as remyelination offers protection against axonal degeneration resulting from demyelination. It is reasonable to posit that a more rapid remyelination process will result in the preservation of a greater number of axons. Chen and colleagues previously demonstrated that in a transgenic murine model in which IFN-γ is secreted by astrocytes in a doxycycline-dependent manner, IFN-γ expression in the CNS suppresses remyelination after cuprizone-induced oligodendrocyte toxicity [182]. Approaches to prolong the ISR pathway, such as Sephin1 treatment or genetic manipulation of GADD34, have been demonstrated to be effective in safeguarding remyelinating oligodendrocytes and promoting the comprehensive restoration of myelin sheaths [182]. In the studied model of inflammatory demyelination and remyelination, the combined treatment with Sephin1 and BZA resulted in a notable increase in myelin thickness without influencing the number of remyelinating oligodendrocytes or the extent of axonal remyelination [182]. In conclusion, the authors posit that the ISR signaling protects remyelinating oligodendrocytes and promotes remyelination in the presence of inflammation, and modulation of the ISR may, therefore, represent an effective neuroprotective therapy for MS patients [180-182].

Guanabenz did not pass the second phase of clinical trials as an anti-ALS drug, primarily due to the adverse effects associated with its α2-adrenergic activity [164]. Sephin1, a derivative of guanabenz, exhibits fewer pronounced side effects and, therefore, merits consideration as a candidate for clinical trials as an anti-ALS drug and a superior analogue of guanabenz. Nevertheless, there are studies that reported the ambiguous role of sephin1. For example, Vieira and colleagues demonstrated that sephin1 delayed the onset of ALS and decreased the ALS-induced upregulation of CHOP, EDEM1, DNAJb9, and HSPA5 but did not affect the expression of the ATF4 gene [115]. The administration of sephin1 to SOD1G93A mice resulted in a notable improvement in the clinical condition [115]. Surprisingly, the authors stated that sephin1 did not produce therapeutic benefits in the SOD1G93A mouse model of ALS [115]. However, this assertion is largely contingent on the hypothesis that sephin1 failed to modulate PERK activity in the ALS model, given that the authors demonstrated that sephin1 did not affect the expression of ATF4 in their study [115]. On this basis, the authors conclude that the beneficial effects of sephin1 are p-eIF2α and PERK-independent [115]. However, this conclusion may be premature, as the authors did not thoroughly assess the activation of the ISR pathway in their study. For instance, they did not evaluate the level of eIF2α phosphorylation in their ALS model [115]. It is noteworthy that the administration of sephin1 to VWM mice did not result in any discernible improvement in motor performance, body weight gain, brain pathology, or suppression of the ATF4-driven transcriptome expression [171]. This contrasts with the observed outcomes following the treatment of VWM mice with guanabenz [171]. In conclusion, sephin1 is a promising modification of guanabenz and merits further investigation as a potential therapeutic agent for a range of neurological disorders.

The beneficial or detrimental effects of ISR activators in various neurological diseases are visualized in Fig. (**3**).

## CONCLUSION

Targeting the Integrated Stress Response has emerged as a promising strategy for managing a variety of neurological disorders. The diverse effects of ISR modulation—whether through inhibitors such as ISRIB, GSK2606414, GSK2656157, and trazodone or activators like salubrinal, sephin1, and guanabenz—highlight the therapeutic potential of fine-tuning this pathway to achieve neuroprotection. Each compound demonstrates unique benefits, mechanisms, and challenges in different disease models, underscoring the complex role of the ISR in neuronal health and disease progression.

The therapeutic modulation of ISR is inherently a double-edged sword, capable of yielding both beneficial and detrimental outcomes depending on the context. While excessive activation of the ISR is implicated in maladaptive processes such as impaired protein synthesis, synaptic dysfunction, and neuronal apoptosis, as seen in AD, ALS, and other proteinopathies, its transient activation is essential for cellular adaptation under acute stress. This dichotomy underscores the need to balance ISR modulation carefully in different disease states.

ISR inhibitors, such as ISRIB, efficiently restore protein synthesis and alleviate cognitive and behavioral deficits in conditions where chronic ISR activation leads to impaired protein translation and synaptic dysfunction. ISRIB, for example, has demonstrated neuroprotective effects across various models, improving cognitive function, enhancing synaptic plasticity, and reducing neuronal loss. It has shown promise in conditions like AD, spinal cord injury, and traumatic brain injury. Notably, in TBI models, the effects of ISRIB persisted well beyond the treatment period, underscoring its potential for durable therapeutic benefits.

In contrast, inhibitors like GSK2606414 and GSK2656157, targeting upstream kinase PERK, can effectively reduce maladaptive ISR signaling but may also induce off-target effects, such as pancreatic toxicity, that limit their clinical applicability. For instance, GSK2606414 has been effective in alleviating ER stress and neuronal apoptosis in models of tauopathy and ischemia, but it has also exacerbated apoptosis and necrosis in ICH models by elevating pro-apoptotic proteins like CHOP and caspase-12. Furthermore, high doses of GSK2606414 have been associated with systemic toxicity, including pancreatic damage and impaired memory functions in certain contexts. Such effects limit their translational potential and highlight the complexity of targeting upstream ISR components, which can affect multiple interconnected pathways. Trazodone, an FDA-approved drug, presents a safer alternative by targeting the ISR indirectly, offering neuroprotective effects without significant adverse outcomes.

On the other hand, ISR activators like salubrinal, sephin1, and guanabenz have shown the potential to protect neurons from acute stress by reducing protein synthesis and enhancing cellular survival mechanisms. These compounds are particularly valuable in scenarios where temporary ISR activation can prevent neuronal damage from misfolded proteins or oxidative stress. Salubrinal has demonstrated neuroprotection in models of TBI and AD by mitigating ER stress and apoptosis, reducing inflammatory markers like TNF-α and IL-6, and promoting cellular membrane integrity. Similarly, guanabenz has shown promise in ALS and AD models by alleviating oxidative stress and improving mitochondrial function.

However, the long-term use of ISR activators must be approached cautiously, as sustained suppression of protein synthesis could hinder normal cellular functions. Guanabenz, for instance, has demonstrated neuroprotective properties but is associated with significant side effects, including α2-adrenergic activity-induced hypotension and impaired glucose tolerance, limiting its therapeutic use. Similarly, sephin1, a guanabenz derivative, has yielded mixed results, showing neuroprotection in some studies but failing to improve outcomes in ALS models.

Overall, these findings highlight the dual nature of ISR modulation in neurological disease treatment. While ISR inhibitors are beneficial in conditions of excessive ISR activation, ISR activators may be more suitable for reducing acute stress and promoting cell survival. The therapeutic potential of ISR modulators appears to depend heavily on the timing and context of their application. Experimental evidence suggests that ISR inhibition is most effective in later stages of chronic neurodegenerative conditions, where translational repression and synaptic dysfunction dominate. In contrast, ISR activation may be beneficial during early stages or acute stress events, helping neurons manage misfolded protein loads and maintain cellular integrity. Notably, some studies highlight contradictory outcomes within the same disease models, likely reflecting differences in ISR modulators’ mechanisms and disease progression stages.

The complex interplay between these approaches suggests that a balanced modulation of the ISR pathway, tailored to specific disease contexts, may offer the most effective therapeutic outcomes. Compounds targeting downstream ISR components, such as ISRIB, may offer greater specificity and fewer side effects compared to upstream inhibitors like GSK2606414, which broadly suppress PERK activity. Conversely, ISR activators must be used with caution, as prolonged suppression of translation can disrupt proteostasis and exacerbate neurodegeneration.

We hypothesize that several approaches could improve the therapeutic application of ISR modulators. For instance, integrating advanced drug design technologies, such as structure-based optimization or high-throughput screening, may identify analogs of existing compounds that exhibit enhanced specificity and reduced side effects. Efforts to optimize the structure of compounds, such as GSK2656157, could mitigate off-target effects like pancreatic toxicity while preserving efficacy, broadening their clinical applicability. Similarly, developing derivatives of activators like guanabenz with reduced α2-adrenergic activity could address side effects like hypotension, as evidenced by advancements with sephin1.

Another strategy lies in refining dosing regimens. High doses of PERK inhibitors like GSK2606414 have been associated with toxicity, while low doses of ISRIB might restore synaptic function without adverse effects such as demyelination. Dose-response studies could reveal optimal therapeutic windows for individual compounds, reducing risks and enhancing benefits.

Timing interventions based on disease progression offers another critical dimension. Activators may be more effective during acute stress to prevent cellular damage, while inhibitors are likely better suited for chronic ISR dysregulation, where restoring protein synthesis and neuronal function is paramount. Modulating specific ISR components, such as ATF4 and CHOP, could enable a more precise therapeutic approach, targeting maladaptive effects while preserving beneficial ISR responses.

Moreover, pairing ISR modulators with anti-inflammatory agents presents a promising combinatory strategy, given the role of neuroinflammation in exacerbating ISR dysregulation. For example, guanabenz’s anti-inflammatory properties could complement ISR inhibition in neurodegenerative diseases such as AD and ALS, amplifying therapeutic benefits.

However, it is important to note that the majority of the studies we describe have been conducted in animal models. There is a great need for randomized controlled trials to assess the efficacy of ISR modulators in humans and to extend the findings to clinical applications in neurological diseases.

To fully realize the potential of ISR-targeting therapies, further research is needed to refine ISR-targeting strategies, optimize safety profiles, and explore combination therapies that leverage both ISR inhibition and activation to address the multifaceted pathology of neurological disorders comprehensively. This nuanced understanding of ISR regulation may pave the way for precision medicine approaches in the treatment of neurodegenerative and neuroinflammatory diseases.

## Figures and Tables

**Fig. (1):**
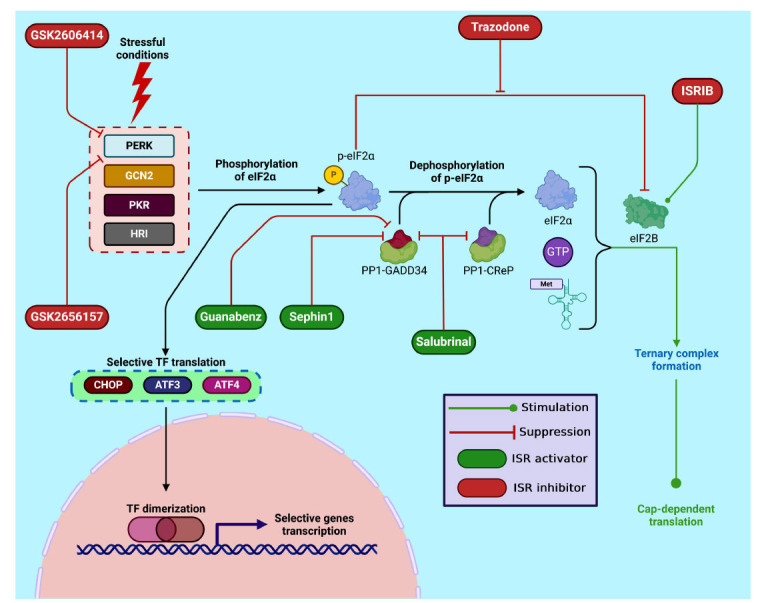
A schematic representation of the ISR pathway and its key pharmacological modulators. Activation of the integrated stress response kinases PERK (endoplasmic reticulum stress), PKR (presence of viral double-stranded RNA), GCN2 (amino acid starvation), HRI (mitochondrial stress) promotes phosphorylating the alpha subunit (eIF2α) of the eukaryotic translation initiation factor, which is essential for the assembly of a ternary complex eIF2-guanosine-5'-triphosphate (GTP)-initiatory Met-tRNAi^Met^ through the guanine nucleotide exchange factor (GEF) eIF2B activity and the translation pre-initiation complex. The phosphorylated alpha subunit of eIF2 blocks the activity of eIF2B and is unable to participate in the ternary complex formation, ultimately reducing cap-dependent translation. Consequently, the selective cap-independent translation of a number of proteins, including the transcription factors ATF3, ATF4, CHOP, and others, occurs when ISR signaling is activated. The dephosphorylation of p-eIF2α is performed after the stress release and catalyzed by protein phosphatase 1 (PP1), which requires the presence of a regulatory subunit, either the constitutive CReP or the stress-inducible GADD34. ISR can also be pharmacologically suppressed by PERK inhibitors GSK2606414 and GSK2656157, as well as by ISRIB (*via* eIF2B activity restoration).

**Fig. (2):**
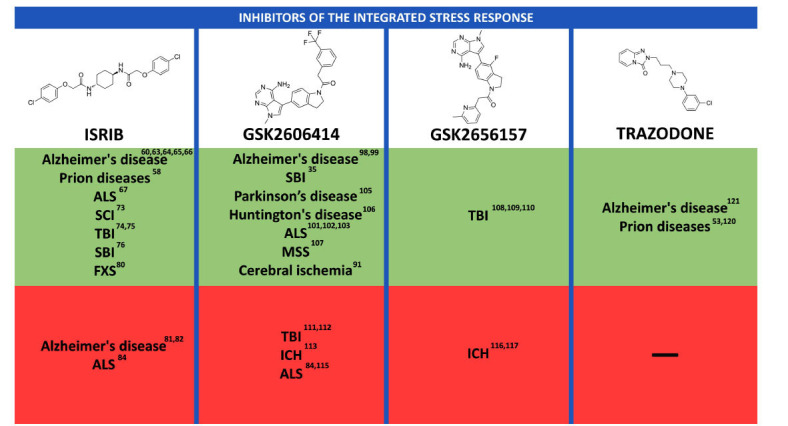
The chemical formulae of the ISR inhibitors are described in the review. Green colors indicate diseases in which the compounds have beneficial effects; red colors indicate undesirable effects or their absence.

**Fig. (3):**
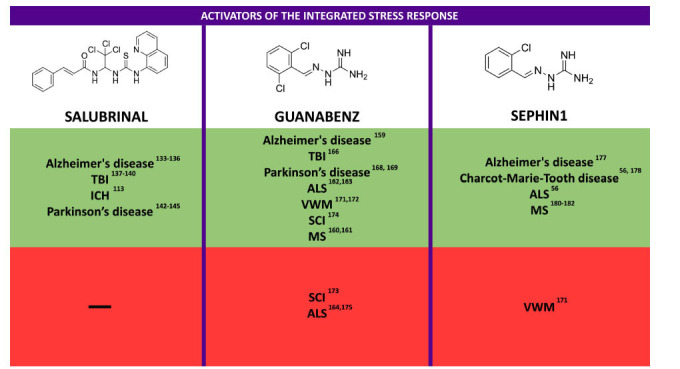
Chemical formulae of the described ISR activators. Abbreviations as in the figure **2**.

## LIST OF ABBREVIATIONS

5-HT: 5-hydroxytryptamine Receptors
6-OHDA: 6-hydroxydopamine
ACM: Astrocyte-conditioned Media
AD: Alzheimer’s Disease
ALS: Amyotrophic Lateral Sclerosis
ALS/FTD: Amyotrophic Lateral Sclerosis and Frontotemporal Dementia
APP: Amyloid-beta Precursor Protein
ARC: Activity Regulated Cytoskeleton Associated Protein
ARF5: ADP-ribosylation Factor 5
ARHGDIA: Rho GDP Dissociation Inhibitor Alpha
ASC: Apoptosis-associated Speck-like Protein Containing a CARD
ASRoD: Age-standardized Rate of Deaths per 100,000 People
ATF: Activating Transcription Factor
ATP5B: ATP Synthase F1 Subunit Beta, Mitochondrial
ATP5J2: ATP Synthase, H+ Transporting, Mitochondrial Fo Complex, Subunit F2
Aβ: Amyloid Beta
BDNF: Brain-derived Neurotrophic Factor
BiP: Binding-immunoglobulin Protein
BZA: Bazedoxifene
bZIP: Basic Leucine Zipper
C/EBP: CCAAT/enhancer-binding Protein
CCND1: Cyclin D1
CDC42: Cell Division Cycle 42
CHI: Closed-head Injury
CHOP: C/EBP Homologous Protein
CMT1B: Charcot-marie-tooth disease type 1B
CNS: Central Nervous System
CRE: Cyclic AMP Response Elements
CREB: cAMP Response Element-binding Protein
CREBZF: CREB/ATF bZIP Transcription Factor
CReP: Constitutive Repressor of eIF2α Phosphorylation
CS: Citrate Synthase
CYC1: Cytochrome C1
DLAT: Dihydrolipoamide S-acetyltransferase
DLG4: Discs Large MAGUK Scaffold Protein 4
DNAJB9: DnaJ Homolog Subfamily B Member 9
DUSP: Dual-specificity Phosphatase
EAE: Experimental Autoimmune Encephalomyelitis
EDEM1: ER degradation-enhancing Alpha-manno-sidase-like 1
eIF2: Eukaryotic Translation Initiation Factor 2
eIF2α: α-subunit of the Eukaryotic Translation Initiation Factor 2
ER: Endoplasmic Reticulum
ERC2: ELKS/RAB6-interacting/CAST Family Member 2
FAD: Flavin Adenine Dinucleotide
FARSB: Phenylalanyl-tRNA Synthetase Beta
FDA: The United States Food and Drug Administration Federal Agency
FMRP: Fragile X messenger Ribonucleoprotein
FOXRED2: FAD Dependent Oxidoreductase Domain Containing 2
FRA1: Fos-related Antigen 1
FXS: Fragile X Syndrome
GADD153: Growth Arrest and DNA Damage-inducible Protein 153
GADD34: Growth Arrest and DNA Damage-inducible Protein 34
GCN2: General Control Nonderepressible-2 Protein Kinase
GluA1: Glutamate Ionotropic Receptor AMPA Type Subunit 1
GluA2: Glutamate Ionotropic Receptor AMPA Type Subunit 2
GPI: Glycosylphosphatidylinositol
GRP78: Glucose-regulated Protein 78
hAPP: Human Amyloid-beta Precursor Protein
HD: Huntington's Disease
HRI: Heme-regulated Inhibitor Protein Kinase
HSPA5: Heat Shock 70 kDa Protein 5
ICH: Intracerebral Hemorrhage
IDH3G: Isocitrate Dehydrogenase (NAD(+)) 3 Non-catalytic Subunit Gamma
IFN: Interferon
IH: Intermittent Hypoxia
IKK: IκB kinase
IL: Interleukin
iNOS: Inducible Nitric Oxide Synthase
IRE: Inositol Requiring Enzyme
IRF3: Interferon Regulatory Factor 3
ISR: Integrated Stress Response
IκB: Inhibitor of Nuclear Factor Kappa B
JNK: C-Jun N-terminal Kinase
L-LTP: Late Long-term Potentiation
LDHA/B: Lactate Dehydrogenase A/B
LFS: Low-frequency Stimulation
LIP: liver-enriched Inhibitory Protein
LPS: Lipopolysaccharides
LTD: Long-term Depression
LTP: Long-term Potentiation
MAF: V-maf Musculoaponeurotic Fibrosarcoma Oncogene Homolog
MAP1B: Microtubule Associated Protein 1B
MAP2: Microtubule-associated Protein 2
MAPK: Mitogen-activated Protein Kinase
Met-tRNAi: Initiator methionyl-tRNA
mGluR5: Metabotropic Glutamate Receptor 5
mOPCs: Mouse Oligodendrocyte Precursor Cells
MS: Multiple Sclerosis
MSS: Marinesco-Sjögren Syndrome
MT-CO2: Mitochondrially Encoded Cytochrome C Oxidase II
mTBI: Mild Traumatic Brain Injury
mtSOD1: Mutant SOD1
NDUFS1: NADH:Ubiquinone Oxidoreductase Core Subunit S1
NE: Neurodegeneration
NF-κB: Nuclear Factor κB
NFE2L1: NFE2 Like bZIP Transcription Factor 1
NLRP3: NLR Family Pyrin Domain Containing 3
NMDAR: N-methyl-D-aspartate Receptor
Nrn: Neuritin
NSF: N-ethylmaleimide-sensitive Factor
OXPHOS: Oxidative Phosphorylation
PAR: Poly(ADP-ribose)
PCs: Purkinje Cells
PCX: Pyruvate Carboxylase
PD: Parkinson’s Disease
PERK: PKR-like Endoplasmic Reticulum Protein Kinase
PFN2: Profilin 2
PKM: Pyruvate Kinase M1/2
PKR: Protein Kinase R
POCD: Postoperative Cognitive Dysfunction
PP1: Protein Phosphatase 1
PSD-95: Postsynaptic Density Protein 95
RAC1: Ras-related C3 Botulinum Toxin Substrate 1
ROS: Reactive Oxygen Species
SBI: Surgical Brain Injury
SCI: Spinal Cord Injury
SDHA: Succinate Dehydrogenase Complex Flavoprotein Subunit A
SKM: Skeletal Muscle
SLC6A11: Solute Carrier Family 6 Member 11
SNARE: Soluble N-ethylmaleimide-sensitive Factor Attachment Protein Receptor
SST: Somatostatin
STING: Stimulator of Interferon Genes
STX1B: Syntaxin 1B
SUCLG1: Succinate-CoA Ligase GDP/ADP-Forming Subunit Alpha
SYN2: Synapsin 2
SYNGAP1: Synaptic Ras GTPase Activating Protein 1
SYNGR1: Synaptogyrin-1
SYP: Synaptophysin
SYT3: Synaptotagmin-3
TBI: Traumatic Brain Injury
TBK1: TANK-binding Kinase 1
TC: Ternary Complex
TCA: Tricarboxylic Acid
TDP-43: Transactive Response DNA Binding Protein 43 kDa
TF: Transcription Factor
TGF: Transforming Growth Factor
Th1: T-helper 1 Cells
TNF: Tumor Necrosis Factor
TPI1: Triosephosphate Isomerase 1
TSC: Tuberous Sclerosis Complex
UCMS: Unpredictable Chronic Mild Stress
uORF: Upstream Open Reading Frame
UPR: Unfolded Protein Response
UQCRC1: Ubiquinol-cytochrome C Reductase Core Protein 1
UQCRC2: Ubiquinol-cytochrome C Reductase Core Protein 2
UTR: 5' Untranslated Region
VAMP3: Vesicle-associated Membrane Protein 3
VWM: Vanishing White Matter
WT: Wild Type
XBP1: X-Box Binding Protein 1
YARS1: Tyrosyl-tRNA Synthetase
αS: α-synuclein
